# RNA-binding protein RPS7 promotes hepatocellular carcinoma progression via LOXL2-dependent activation of ITGB1/FAK/SRC signaling

**DOI:** 10.1186/s13046-023-02929-1

**Published:** 2024-02-08

**Authors:** Yu-Jiao Zhou, Min-Li Yang, Xin He, Hui-Ying Gu, Ji-Hua Ren, Sheng-Tao Cheng, Zhou Fu, Zhen-Zhen Zhang, Juan Chen

**Affiliations:** 1 Department of Infectious Disease, Children’s Hospital of Chongqing Medical University, National Clinical Research Center for Child Health and Disorders, Ministry of Education Key Laboratory of Child Development and Disorders, Chongqing Key Laboratory of Child Rare Diseases in Infection and Immunity, No.20 Jinyu Road, Yubei District, Chongqing, 401122 China; 2 The Key Laboratory of Molecular Biology of Infectious Diseases designated by the Chinese Ministry of Education, Chongqing Medical University, Chongqing, China; 3 Stem Cell Biology and Therapy Laboratory, Ministry of Education Key Laboratory of Child Development and Disorders, and the Department of Respiratory Diseases, The Children’s Hospital of Chongqing Medical University, Chongqing, China; 4 Key Laboratory of Laboratory Medical Diagnostics, Chinese Ministry of Education, Chongqing Medical University, No.1 Youyi Road, Yuzhong District, Chongqing, 400016 China

**Keywords:** Hepatocellular carcinoma, RNA-binding protein, Ribosomal protein S7, Lysyl oxidase-like 2, Focal adhesion, Metastasis

## Abstract

**Background:**

Metastasis is one of the leading cause contributes to treatment failure and poor prognosis of hepatocellular carcinoma (HCC) patients. The underlying mechanism of HCC metastasis remains to be determined. Although several RNA binding proteins (RBPs) have been found to participate in tumorigenesis and progression of liver cancer, the role of RBPs in HCC patients with extrahepatic metastases is poorly understood.

**Methods:**

By performing RNA-seq of primary HCC tissues (including HCC with extrahepatic metastasis and those did not develop metastasis), we identified a set of HCC metastasis-associated RBPs candidates. Among which, ribosomal protein S7 (RPS7) was found to be remarkably increased in HCC tissues and be strongly related to HCC poor survival. Overexpression or CRISPR-Cas9–mediated knockout were applied to investigate the role of RPS7 on the metastasis-associated phenotypes of HCC cells. RNA sequencing, RIP, RNA-pull down, dual luciferase reporter assay, nascent RNA capture assay, and RNA decay and so on, were applied to reveal the underlying mechanism of RPS7 induced HCC metastasis.

**Results:**

Gain- and loss- of function analyses revealed that RPS7 promoted HCC cells adhesion, migration and invasion capabilities, as well as lung metastasis. Mechanistically, we uncovered that lysyl oxidase-like 2 (LOXL2) was a critical downstream target of RPS7. RPS7 could stabilize LOXL2 mRNA by binding to AUUUA motifs in the 3155–3375 region of the 3’UTR of LOXL2 mRNA, thus increased LOXL2 expression via elevating LOXL2 mRNA abundance. Further research revealed that LOXL2 could accelerate focal adhesion formation through maintaining the protein stability of ITGB1 and activating ITGB1-mediated FAK/SRC signaling pathway, and thereby contribute to the pro-metastasis effect of RPS7.

**Conclusions:**

Taken together, our data reveal a novel function of RPS7 in HCC metastasis, also reveal the critical roles of the RPS7/LOXL2/ITGB1 axis in HCC metastasis and shed new light on the exploration of molecular drugs against HCC.

**Supplementary Information:**

The online version contains supplementary material available at 10.1186/s13046-023-02929-1.

## Background

According to the 2020 global cancer report, hepatocellular carcinoma (HCC) ranks sixth in incidence but third in mortality among all malignancies [1]. Metastasis is one of the leading causes contributing to treatment failure and the low survival rate of HCC patients [2]. Although the existing molecular targeted drugs (sorafenib, etc.) can prolong life for patients with advanced liver cancer to some extent, they are incapable of effectively preventing recurrence and metastasis [3]. Thus, further understanding the molecular mechanisms underlying HCC metastasis and exploring novel therapeutic targets for HCC treatment are of paramount importance.

RNA-binding proteins (RBPs) are an important class of proteins in cells that recognize and bind RNAs via some specific RNA-binding domains, widely participate in various posttranscriptional regulatory processes, including splicing, localization, degradation, modification and translation of cellular RNAs, in turn, regulate the expression of some specific genes [4–6]. Recently, the role of the RBP-mediated gene expression regulatory network in the occurrence of cancers has gradually attracted the attention of researchers. Although over 1500 RBPs have been identified in the human genome, how and to what extent RBPs modulate the cancer transcriptome remains largely unexplored. Ribosomal proteins (RPs) are a family of canonical RBPs. In addition to playing central roles in ribosome biogenesis and protein synthesis, RPs are also found to be involved in cell proliferation, apoptosis, invasion and metastasis via a number of mechanisms including transcription regulation, autophagy, phosphorylation and RNA-binding function [7–10], and therefore participate the occurrence and development of multiple cancers types. Among RPs, human ribosomal protein S7 (RPS7) is receiving increasing attention for its different functions in multiple cancers. RPS7 functions as a tumor suppressor in colorectal cancer and ovarian tumors, whereas it acts as a pro-oncogenic factor in prostate cancer, lung adenocarcinoma and breast cancer [11–15]. Nonetheless, the role of RPS7 in the occurrence and development of HCC remains unclear.

In this study, we identified that RPS7 was markedly upregulated in HCC tissues with extrahepatic metastasis compared to that in metastasis-free HCC tissues. According to mechanisms studies, we identified that LOXL2-mediated activation of ITGB1/FAK/SRC signaling pathway was involved in RPS7-induced HCC metastasis. Collectively, our findings suggest that RPS7 has potential as a novel therapeutic target for HCC treatment.

## Materials and methods

### Cell culture

The human liver cancer cell lines MHCC97H and HLE were obtained from the Liver Cancer Institute of Zhongshan Hospital of Fudan University (Shanghai, China). Huh7 and PLC/PRF/5 cells were purchased from the Health Science Research Resource Bank. Cells were cultured in Dulbecco’s modified Eagle’s medium (DMEM) supplemented with 10% fetal bovine serum (Gibco) and 1% penicillin/streptomycin and incubated at 37 °C with 5% CO2. Cell line authentication was carried out by Guangzhou Jennio Biotech Co., Ltd., Guangzhou, China.

For further details regarding the materials and methods used, please refer to supplementary information.

## Results

### Identification of RPS7 as the key RBP associated with HCC metastasis

**Fig. 1:**
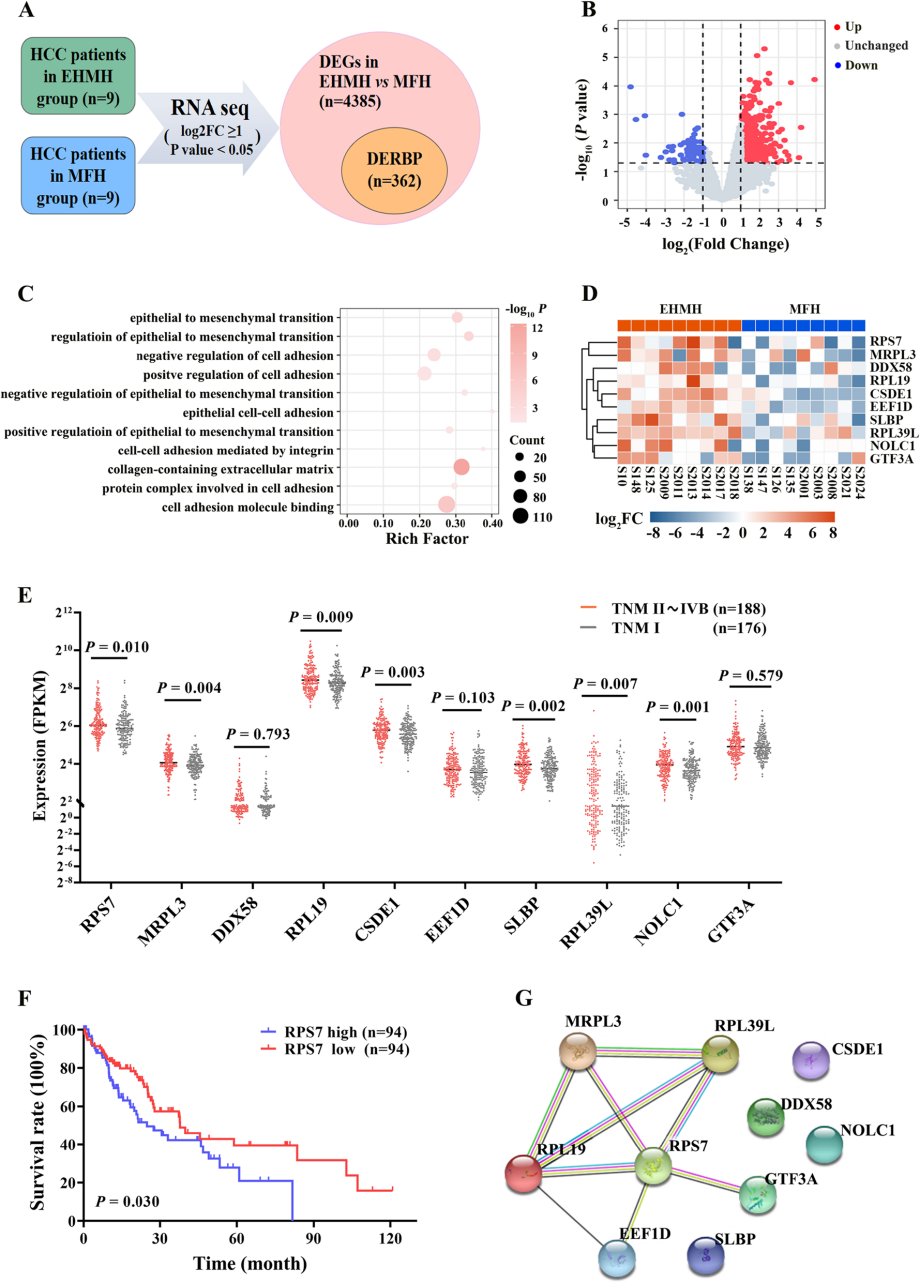
Identification of RPS7 as an important RNA-binding protein (RBP) that associates with HCC progression. Nine pairs of primary HCC tissues with extrahepatic metastasis (EHMH) and nine pairs of metastasis-free HCC tissues (MFH) as well as corresponding adjacent nontumoral tissues (ANT) were used for RNA sequencing analysis, aiming to screen and identify RBP that significantly correlate with HCC metastasis. The clinical data of HCC patients from TCGA-LIHC datasets were used for validation. According to the tumor-node-metastasis (TNM) staging system for liver cancer, patients were classified into metastasis group (TNM II~IVB) and metastasis-free group (TNM I ). **A** Schematic screening workflow of differentially expressed RBPs (DERBPs) in EHMH compared to MFH. **B** Volcano plot for DERBPs expression. **C** Go analysis of 312 upregulated DERBPs. **D** Heat map for the top 10 upregulated DERBPs. **E** The expression difference of each gene was analyzed by comparing metastasis group with metastasis-free group based on TCGA database. **F** The correlation between RPS7 levels and overall survival rate in HCC with metastasis. **G** The PPI network by String database

To identify HCC metastasis-associated RBP, we performed RNA sequencing (RNA-seq) in 9 pairs of primary HCC tissues with extrahepatic metastasis (EHMH) and 9 pairs of metastasis-free HCC tissues (MFH). In total, 5,149 differentially expressed genes (DEGs) were found in EHMH and 4,087 DEGs was found in MFH, compared with paired adjacent nontumoral tissues (ANT) respectively. We further found that 4385 DEGs in EHMH compared to MFH, of which 3867 genes were up-regulated and 518 genes were down-regulated (log2FC ≥ 1; *P* value < 0.05). A total of 362 RNA-binding protein genes were found dysregulated in EHMH compared to MFH, of which 312 genes were up-regulated and 50 gene was down-regulated (log2FC ≥ 1; *P* value < 0.05) (Fig. 1A and B). The GO enrichment analysis revealed that 312 upregulated RBP genes were mainly enriched in multiple metastasis processes, such as cell adhesion, epithelial to mesenchymal transition and collagen − containing extracellular matrix (Fig. 1C). The top 10 upregulated RBP genes were RPS7, MRPL3, DDX58, RPL19, CSDE1, EEF1D, SLBP, RPL39L, NOLC1 and GTF3A (Fig. 1D).

Next, we extracted clinical data of HCC patients from TCGA-LIHC datasets (https://tcga-data.nci.nih.gov/tcga/). According to the tumor-node-metastasis (TNM) staging system, patients were divided into two groups, including 188 HCC with intra- and/or extra-hepatic metastases (TNM II~IVB group) and 176 HCC without metastases (TNM I group). We then analyzed the expression differences of the 10 candidates between the two groups, and revealed that 7 genes including RPS7, MRPL3, RPL19, etc., were significantly highly expressed in the metastasis group compared with the metastasis-free group (Fig. 1E). Next, by generating Kaplan-Meier survival curves, we found that only RPS7 and MRPL3 presented a remarkable negative correlation with the overall survival (OS) rate in the patients with metastasis, respectively (Fig. 1F; Fig. S1A). Furthermore, a PPI network among the above 10 candidate genes was performed by STRING database, and RPS7 was found to be maintained in the core position (Fig. 1G). These data indicated that RPS7 might be the most discriminating candidate that correlated with HCC metastasis. Additionally, based on TCGA datasets, we also found that the expression level of RPS7 has no significant differences compared to normal tissues in some common malignancies, such as lung cancer, colon cancer, breast cancer and prostate cancer (Fig. S1B), implying that differentially RPS7 expression is HCC specific. Additionally, with the use of the UALCAN online database (http://ualcan.path.uab.edu/index.html), we found that RPS7 expression was significantly associated with histological grades and clinical stages in HCC patients (Fig. S1C). Collectively, these data imply that RPS7 is a key RBP that closely correlates with HCC poor prognosis.

#### High levels of RPS7 were associated with metastasis and poor prognosis of HCC patients

**Fig. 2:**
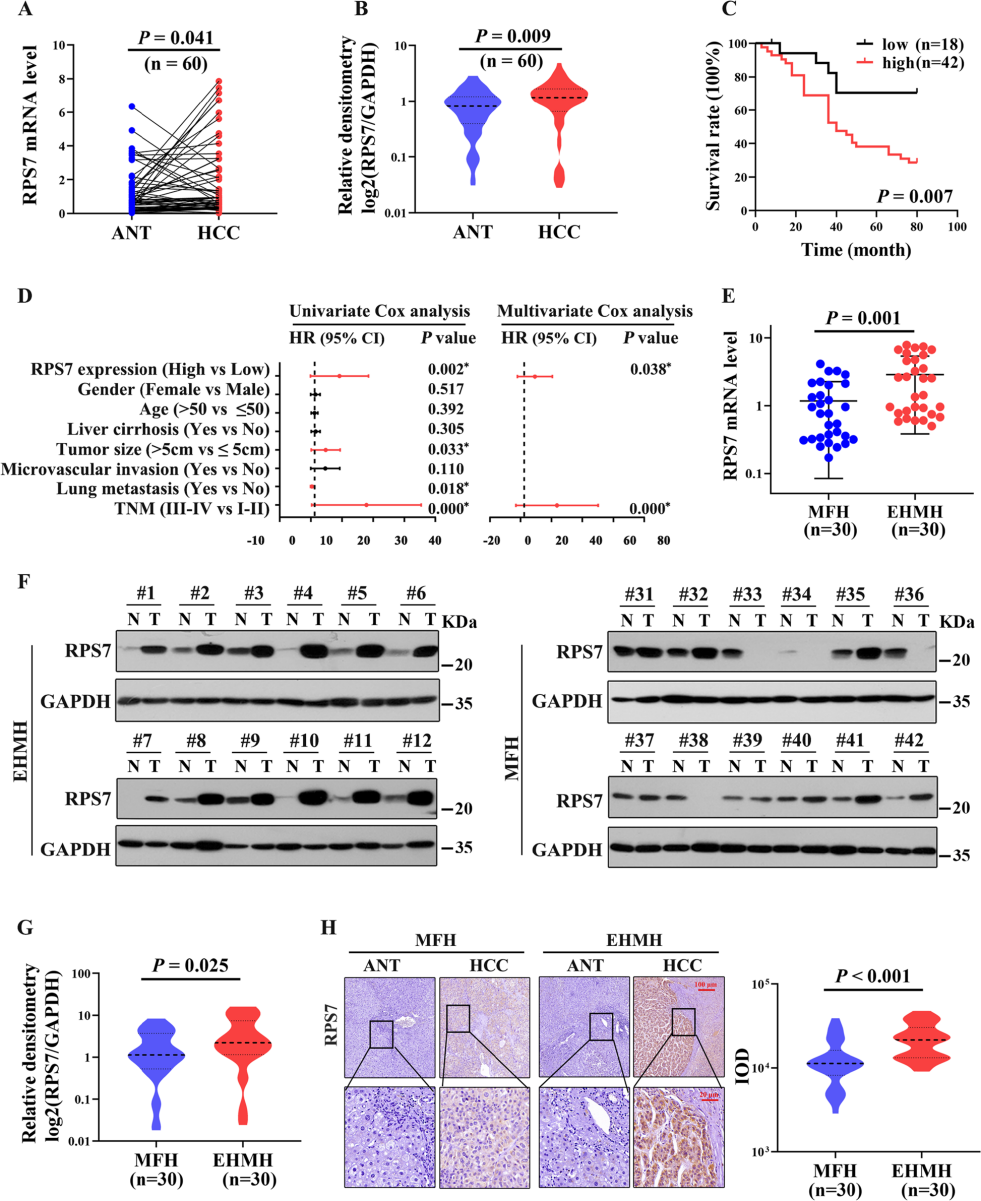
RPS7 is up-regulated in HCC with metastasis and is correlated with poor survival. Sixty pairs of HCC samples and matched adjacent nontumoral tissues (ANT) were used to detect the expression of RPS7. Among which, thirty pairs were tissues came from HCC patients with extrahepatic metastasis and other 30 pairs were from metastasis-free HCC patients, designated as EHMH and MFH, respectively. **A** The mRNA levels of RPS7 in HCC tissues compared to matched ANT. **B** The protein levels of RPS7 in HCC tissues compared to matched ANT. **C** The correlation between RPS7 levels and HCC overall survival was evaluated by KM curve. **D** Forest plot represented the correlation between RPS7 expression and HCC survival, which was evaluated by using univariate and multivariate Cox regression analyses. ^*^, *P *< 0.05. **E** The comparison of RPS7 mRNA levels between EHMH group and MFH group. **F** RPS7 protein levels in EHMH and MFH were respectively detected by western blot assay, as shown was the representative result. **G** The comparison of RPS7 protein levels between EHMH group and MFH group. H. The expression and localization of RPS7 in HCC tissues compared to matched ANT were analyzed by Immunohistochemistry

To clarify the clinical significance of RPS7 in human HCC, we examined RPS7 expression in 60 pairs of primary HCC and adjacent nontumoral liver tissues collected in our laboratory, in which 30 pairs were HCC tissues with metastasis (EHMH), other 30 pairs were metastasis-free HCC tissues (MFH). Our results showed that compared to adjacent nontumorous tissues (ANT), mRNA and protein levels of RPS7 were significantly upregulated in HCC tissues (Fig. 2A and B). There was a significant negative relationship between RPS7 expression levels and HCC prognosis (Fig. 2C).

We then analyzed the correlation between RPS7 levels and clinicopathologic characteristics in these patients. As shown in Table 1, RPS7 expression levels were significantly associated with microvascular invasion, lung metastasis and TNM stage, whereas no significant association was found between its expression levels and sex, age, AFP level, liver cirrhosis or tumor size. Moreover, according to univariate and multivariate Cox regression analyses, we further identified that RPS7 has potential to be an independent predictor of the survival of HCC patients in addition to TNM stage (Fig. 2D).

**Table 1:** Correlation between clinicopathological characterastics and RPS7 expression of 60 HCC patients

characteristics	Numbers	Expression of RPS7		***p-value***
		High (*n*=42)	Low (*n*=18)	
**Gender**				
male	48	33	15	0.999
female	12	9	3	
**Age**				
≤50	24	15	9	0.391
>50	36	27	9	
**AFP(ug/mL)**				
≤400	41	31	10	0.227
>400	19	11	8	
**Liver cirrhosis**				
positive	41	28	13	0.768
negative	19	14	5	
**Tumor size**				
≤5cm	25	16	9	0.409
>5cm	35	26	9	
**Tumor number**				
single	54	39	15	0.352
multiple	6	3	3	
**Encapsulation**				
complete	38	30	8	0.078
no	22	12	10	
**PVTT**				
yes	11	8	3	0.999
no	49	34	15	
**Microvascular invasion**				
yes	33	27	6	0.046*
no	27	15	12	
**Lung etastasis**				
yes	19	17	2	0.034*
no	41	25	16	
**TNM stage**				
I and II	25	13	12	0.021*
III and IV	35	29	6	

Chi square test was used. AFP, α-fetoprotein; PVTT, Portal vein tumor thrombosis. *p-value< 0.05

To further determine the role of RPS7 in HCC metastasis, we compared the expression levels of RPS7 between EHMH and MFH groups. Our data showed that mRNA levels of RPS7 in the EHMH group were markedly higher than those in the MFH group (Fig. 2E). Consistently, similar results were found on protein levels of RPS7 in HCC tissues by using western blot assay and IHC analysis (Fig. 2F-H, Fig. S2). Conclusively, these results indicate that high levels of RPS7 clearly associate with metastasis and poor outcomes in HCC patients.

### RPS7 promotes HCC cell proliferation, adhesion, migration and invasion

**Fig. 3:**
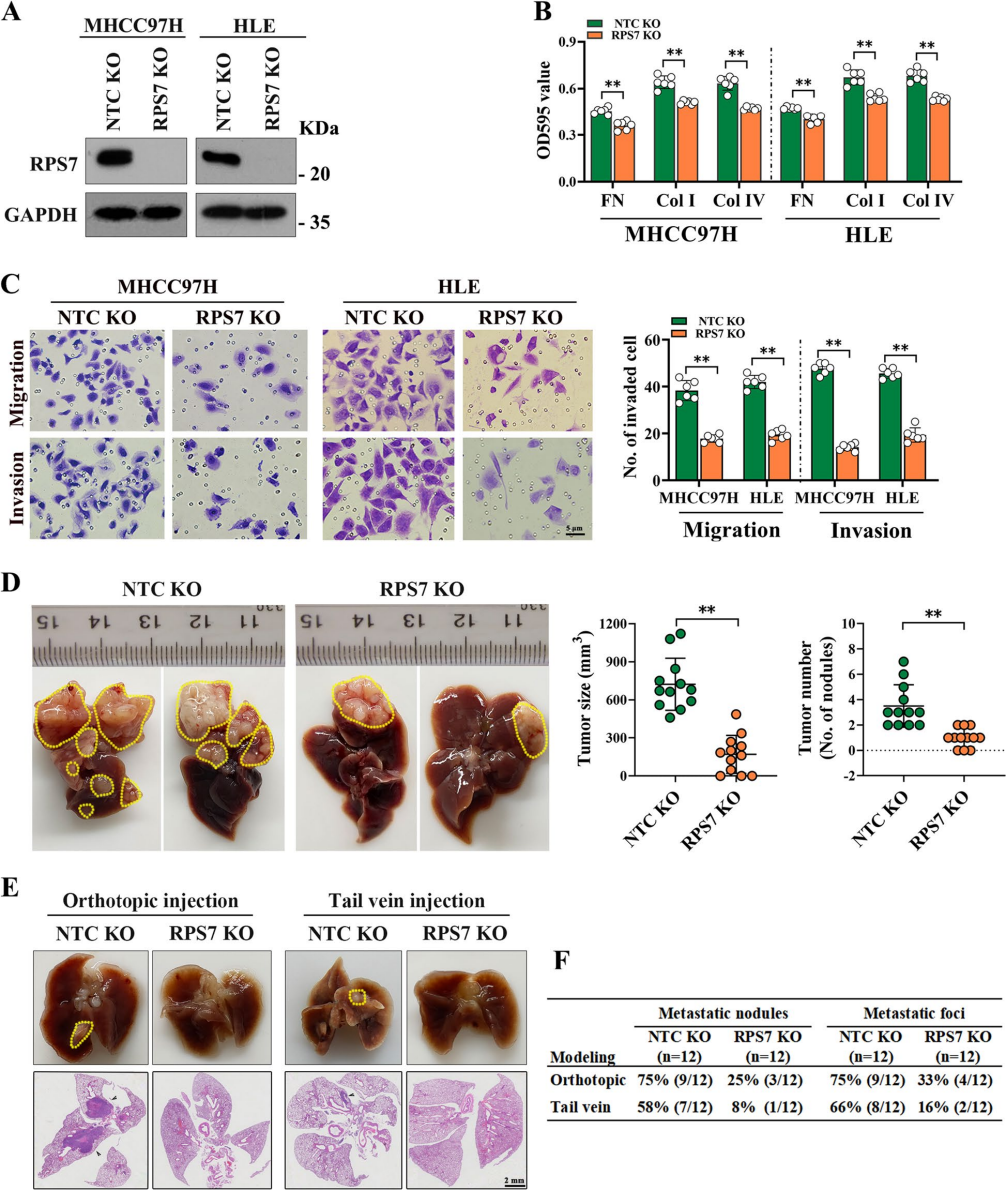
RPS7 knockout inhibits HCC cell adhesion, migration and invasion in vitro and metastasis in vivo. MHCC97H and HLE cells, two highly invasive HCC cell lines were used to establish stable RPS7 knockout cells by using CRISPR/Cas9 system (RPS7 KO). Non-target knockout cells (NTC KO) were correspondingly used as controls. The cell-matrix adhesion capacity, migration and invasion ability of cells in vitro as well as metastasis in vivo were observed. **A** The knockout efficiency was determined by western blot assay. **B** RPS7-knockout MHCC97H and HLE cells adhesion to fibronectin, collagen I and collagen IV were detected using cell-matrix adhesion assay. **C** The effect of RPS7-knockut on cell migration and invasion were determined by Transwell assay. **D** Orthotopic mouse models were constructed using RPS7-knockout MHCC97H cells and control cells (each group, *n*=12). The effect of RPS7-knockout on tumor size and numbers were evaluated. **E** and **F** The effects of RPS7-knockout on lung metastasis were evaluated by orthotopic mouse models and tail vein lung metastasis mouse models, respectively. Representative data are from at least 3 independent experiments. Data are shown as mean ± SD. **, *P* < 0.01

To investigate the function of RPS7 in HCC cells, MHCC97H and HLE, two highly aggressive HCC cell lines, were used to establish stably RPS7-knockout cell lines by using CRISPR/Cas9 system (Fig. 3A). According to CCK-8 assay and colony-formation assay, we found that RPS7 knockout resulted in a significant reduction of proliferation and colony-formation abilities in HCC cells (Fig. S3A, S3B). In contrast, over-expression of RPS7 (Fig. S4A) markedly enhanced cell proliferation and colony formation abilities, which was found in Huh7 and PLC/PRF/5, two low-aggressive HCC cell lines (Fig. S3C, S3D). Given the fact that adhesion of cancer cells to extracellular matrix (ECM) is the first step of metastasis [16], three different components of the ECM, collagen I (Col I), collagen IV(Col IV), and fibronectin (FN), were used to evaluate the adhesion capacity of designated HCC cells. A remarkable decreased cell adhesion to Col I, Col IV and FN were observed in RPS7-knockout cells (Fig. 3B), while opposite results were observed in RPS7 over-expressing cells (Fig. S4B). Moreover, RPS7 knockout markedly diminished the wound-healing, migration and invasion abilities of MHCC97H and HLE cells (Fig. S3E; Fig. 3C), whereas RPS7 overexpression enhanced the wound-healing, migration and invasion abilities of Huh7 and PLC/PRF/5 cells (Fig. S4C, S4D).

To further assess the effect of RPS7 on tumor metastasis in vivo, two types of mouse models of HCC lung metastasis were constructed by orthotopic transplantation and tail vein injection, respectively. RSP7 knockout cells or negative control cells were orthotopically injected into the left lobe of orthotopic liver of the nude mice. In this mouse model, RPS7 knockout dramatically suppressed tumor size and tumor number compared to controls (Fig. 3D). With respect to the lung metastasis, about 75% (9/12) of control mice developed lung metastases, while only 25% (3/12) of RPS7-knockout mice had lung metastases. On the other hand, RSP7 knockout cells or negative control cells were injected via tail vein. Ten weeks after injection, 58% (7/12) of the control groups exhibited lung metastatic nodules, whereas 8% (1/12) visible metastatic nodules were found in RPS7-knockout group. Similar results were observed using microscopy (metastatic foci) (Fig. 3E and F). In contrast, RPS7 over-expressed Huh7 cells resulted in larger tumor size and more tumor nodules, as well as increased lung metastasis compared to the controls (Fig.S4E-G). Taken together, these findings demonstrate that RPS7 promotes HCC cells growth, adhesion, migration and invasion.

### RPS7 binds to LOXL2 mRNA and increases its stability

**Fig. 4:**
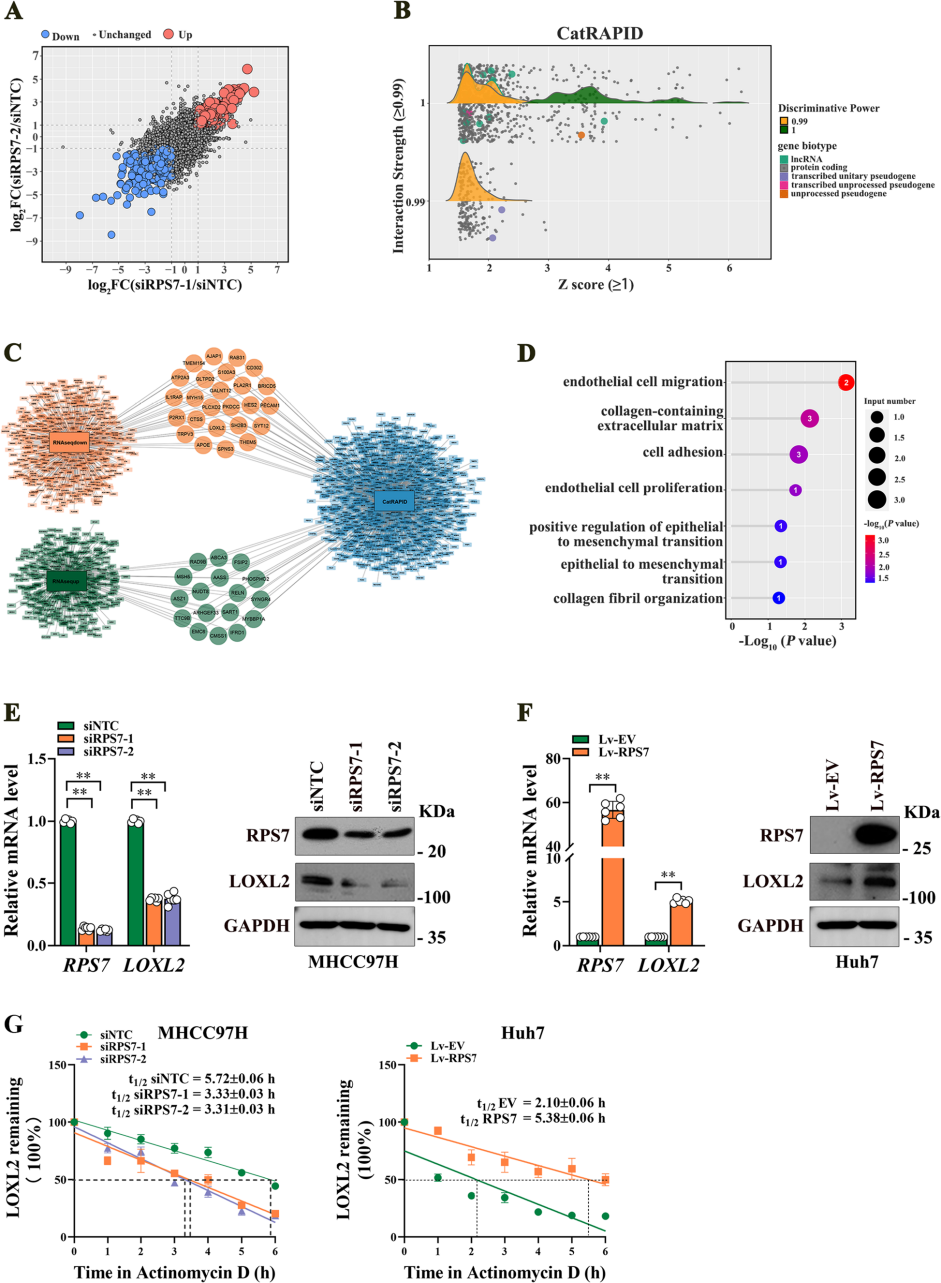
LOXL2, a key downstream target of RPS7, is regulated by RPS7 at post-transcriptional level. To elucidate the potential mechanism underlying RPS7-mediated HCC metastasis, RAN-seq analysis was performed using two independent RPS7-knockdown (siRPS7-1 and siRPS7-2) and non-target knockdown (siNTC) MHCC97H cells. **A** As shown were differentially expressed genes after RPS7 knockdown. **B** Candidate transcripts that may bind to RPS7 protein were extracted from catRAPID database. **C** After combing the RNA-seq data and catRAPID data set, differentially expressed genes that possibly bind to RPS7 protein were identified. **D** KEGG analysis of the candidate downstream genes. **E** RPS7 knockdown decreased the mRNA and protein levels of LOXL2. F. RPS7 overexpression increased the mRNA and protein levels of LOXL2. G. The effect of RPS7 on LOXL2 mRNA half-life was determined by RNA decay assay. **, *P* < 0.01

To explore the molecular mechanism underlying the pro-metastatic effect of RPS7 on HCC, we performed RNA-Seq analysis between RPS7 knockdown cells and control parental cells. A total of 898 differentially expressed genes were independently regulated by RPS7, including 350 consistently up-regulated genes and 548 consistently down-regulated genes (Fig. 4A). Due to RPS7 is one kind of RBP, we further utilized the catRAPID database, an algorithm to estimate the binding propensity of protein-RNA pairs, to predict the RNAs that may bind to RPS7 protein. And 1069 protein-coding transcripts were identified (Fig. 4B). We intersected these two datasets and found that 42 differentially expressed genes possibly bind to RPS7 protein, of which 25 genes were down-regulated and 17 genes were up-regulated (Fig. 4C). The KEGG pathway analysis further showed that six RPS7-regualted genes including LOXL2, PECAM1, CTSS, APOE, RELN and AJAP1, were mainly involved in process of metastasis (Fig. 4D). Interestingly, LOXL2 was found to be participated in all the indicated pathways, indicating a strong correlation between LOXL2 and HCC metastasis.

**Fig. 5:**
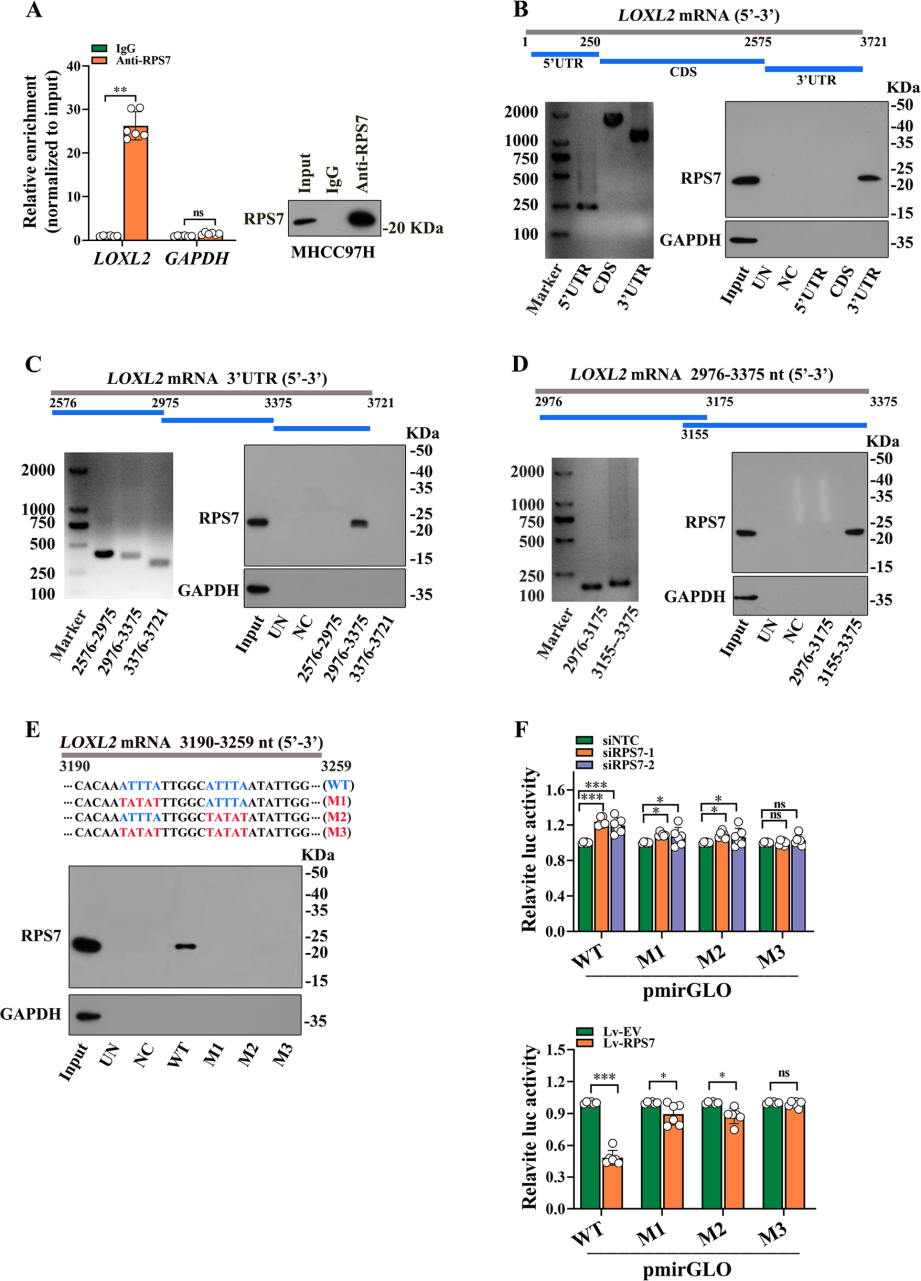
RPS7 regulates LOXL2 expression via binding to LOXL2 mRNA. **A** RNA immunoprecipitation assay was performed in MHCC97H cells by using antibody targeting RPS7, qRT-PCR was performed to detect the mRNA level of LOXL2 in the precipitated RNA-Protein complex. **B**-**E** To further identify the specific binding region of RPS7 on LOXL2 mRNA, a series of RNA pull-down analyses were performed using different biotinylated fragments of LOXL2 mRNA including 5’UTR, CDS, 3’UTR (**B**), and 2576-2975 nt, 2976-3375 nt, 3376-3721 nt (**C**), 2976-3175 nt, 3155-3375 nt (**D**) within 3’UTR, as well as truncated recombinants of 3190-3259 nt (**E**). Western blot assay was subsequently used to detect RPS7 in these indicated mRNA fragments pull-down complex. Input showed 1% lysate. UN represented a control using beads only. NC represented a negative control using non-blot RNA. F. The pmirGLO-derived luciferase reporter containing wild-type and three different mutants of AUUUA motifs were constructed, 293T cells were transfected with these plasmids respectively. Luciferase activities normalized against Renilla luciferase activities were measured to determine the binding efficient of the AUUUA motifs to RPS7 in response to RPS7 overexpression or RPS7 knockdown. *, *P* < 0.05, **, *P* < 0.01, ***, *P* < 0.001. ns, no significant

Next, we performed real-time PCR and western blot analysis to validate that RPS7 knockdown resulted in markedly decreased mRNA and protein levels of LOXL2 in HCC cells, while RPS7 overexpression promoted expression of LOXL2 (Fig. 4E and F). We then performed experiments to elucidate the decreased LOXL2 mRNA was due to a change in RNA synthesis or RNA decay. In nascent RNA capture assays, the rate of LOXL2 mRNA remained unchanged after RPS7 knockdown or overexpression (Fig. S5A). Consistently, luciferase reporter assay found that RPS7 silencing or overexpression did not affect promoter activity of LOXL2 gene (Fig.S5B). We then evaluated whether RPS7 could stabilize LOXL2 mRNA by the treatment with actinomycin D, an inhibitor of RNA synthesis. The half-life of LOXL2 mRNA was decreased by RPS7 silencing after actinomycin D treatment, whereas RPS7 overexpression resulted in increased half-life of LXOL2 mRNA (Fig. 4G), suggesting that RPS7 maintains LOXL2 mRNA stability. Results of subsequent RNA immunoprecipitation (RIP) assay revealed that LOXL2 mRNA was remarkably enriched in RPS7-immuneprecipitates, compared with the amount of GAPDH mRNA (Fig. 5A). To further determine the specific binding region of RPS7 on LOXL2 mRNA, we divided LOXL2 mRNA into different fragments, then, performed RNA pull-down experiments using biotin-labeled LOXL2 mRNA fragments. Our data found that RPS7 interacted with the 3’UTR of LOXL2 mRNA, but not the 5’UTR or CDS (Fig. 5B). We further divided the 3’UTR of LOXL2 mRNA into three fragments and found that RPS7 mainly interacted with the 2976–3375 fragment, of which 3155–3375 fragment was the main binding site of RPS7(Fig. 5C and D). Moreover, two consecutive AUUUA motifs, which belong to the AU-rich element (a common RBP-binding site), were found within the 3155–3375 fragment. To investigate whether the AUUUA motifs were essential for the interaction of LOXL2 mRNA with RPS7, we generated M1 and M2 mutant in which single motif was mutated respectively, and M3 in which both AUUUA motifs were mutated. Compared with the wild type (WT) fragment, each of the mutants lost its ability to bind to RPS7 (Fig. 5E). Moreover, WT fragment, M1, M2 and M3 mutant were separately cloned into the pmirGLO vector to confirm whether RPS7-mediated LOXL2 mRNA stability was dependent on AUUUA motifs within the 3155–3375 fragment. RPS7 deletion resulted in most significantly increased luciferase activity in pmirGLO-WT group, compared to pmirGLO-M1, pmirGLO-M2 and pmirGLO-M3. In contrast, most significantly decreased luciferase activity of pmirGLO-WT was displayed in response to RPS7 overexpression (Fig. 5F). Collectively, these data suggest that RPS7 directly binds to 3’UTR of LOXL2 mRNA to increase its stability, two AUUUA motifs in 3’UTR 3155–3375 fragment are essential for interaction between RPS7 and LOXL2 mRNA.

#### LOXL2 promotes malignant phenotypes of HCC cells

**Fig. 6:**
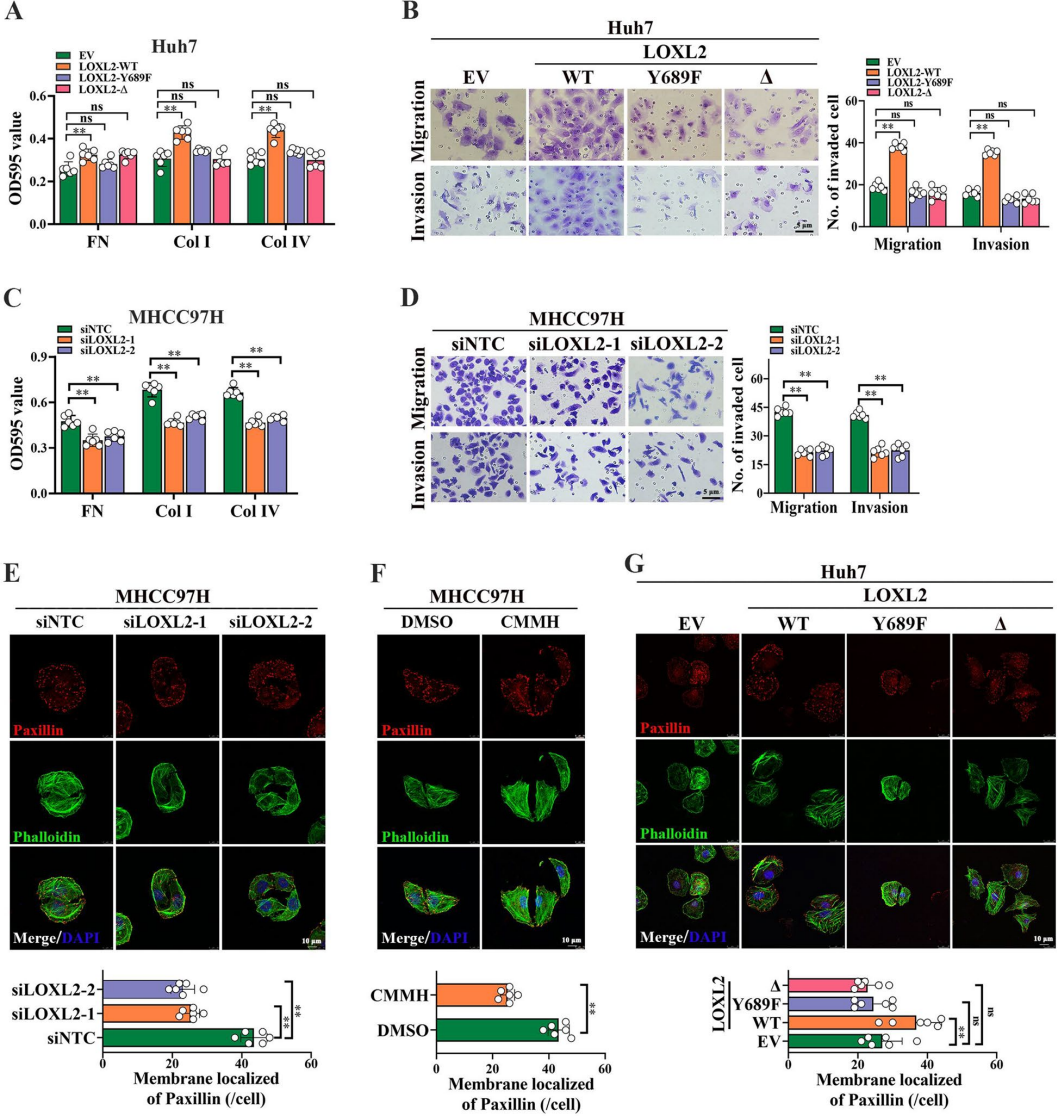
LOXL2 promotes focal adhesion (FA) formation, migration and invasion of HCC cells. **A** and **B** To evaluate the role of LOXL2 in HCC metastasis, two pcDNA3.1-LOXL2-3×Flag mutant plasmids, catalytic activity deletion mutant of LOXL2 (LOXL2-Δ) and catalytically inactive point mutant of LOXL2 (LOXL2-Y689F), as well as wild type LOXL2 (LOXL2-WT) were respectively constructed and stably transfected into Huh7 cells. The effect of LOXL2 overexpression on cell-matrix adhesion ability (**A**) and migration and invasion (**B**) were analyzed. **C** The effect of LOXL2 silencing on cell-matrix adhesion ability in MHCC97H cells. **D** The effect of LOXL2 silencing on cell migration and invasion in MHCC97H cells. **E**-**G**. The effect of LOXL2 silencing (**E**), 20 μM CMMH (**F**), and LOXL2 overexpression (**G**) on FA formation in HCC cells were respectively detected by Immunofluorescence staining experiments. Representative data are from at least 3 independent experiments. Data are shown as mean ± SD. **, *P* < 0.01. ns, no significant

To investigate the importance of LOXL2 in metastasis process of HCC, two mutant plasmids, catalytic activity deletion mutant of LOXL2 (LOXL2-Δ) and catalytically inactive point mutant of LOXL2 (LOXL2-Y689F), were respectively constructed based on pcDNA3.1-3×Flag vector and stably transfected into Huh7 cells (Fig. S6A, S6B). Overexpression of wild type LOXL2 (LOXL2-WT), but not the LOXL2-Δ or LOXL2-Y689F, caused an enhancement of the cell-matrix adhesion capacity, as well as the migration and invasion capabilities of Huh7 cells (Fig. 6A and B). In contrast, LOXL2 knockdown (Fig. S6C) or (2-Chloropyridin-4-yl)MethanaMine Hydrochloride (CMMH) with dose of 20 µM (Fig. S6D), a highly selective LOXL2 enzyme inhibitor, respectively resulted in significantly decreased cell-matrix adhesion capacity, migration and invasion capabilities of MHCC97H cells (Fig. 6C and D; Fig. S6E,S6F). Importantly, IF assay analysis of Paxillin, an integrin-associated protein of FA plaques, found that LOXL2 knockdown or CMMH treatment markedly reduced FA amounts localized on membrane of MHCC97H cells (Fig. 6E F). In contrast, overexpression of LOXL2-WT enhanced the number of FA (Fig. 6G).

To investigate whether LOXL2 was involved in RPS7-induce HCC metastasis, we treated Huh7 cells overexpressing RPS7 with CMMH. 20 µM CMMH markedly blocked the RPS7-induced enhancement of cell-matrix adhesion, migration and invasion capabilities of HCC cells (Fig. S6G, S6H). Moreover, we overexpressed LOXL2 in RPS7-knockout MHCC97H cells, found that the adhesion, migration and invasion abilities of RPS7 knockout cells were significantly restored by LOXL2 overexpression (Fig. S6I, S6J).

We further conducted animal experiments. On the one hand, RPS7-overexpressed Huh7 cells were used to establish orthotopic HCC models as previously described. Four weeks after inoculation, CMMH (15 mg/kg) was administered twice a week for six weeks through tail vein injection. As the results showed, expression of LOXL2 in tumor tissues in CMMH-treated mice were downregulated compared to those in PBS group (Fig. S7A). CMMH intervention did not cause significant changes in the size and number of orthotopic liver tumors, however, numbers of lung metastases in CMMH-treated mice were fewer than those in PBS-treated group (Fig. S7B-D). On the other hand, orthotopic HCC models were established by using RPS7-deletion MHCC97H cells. At 2 weeks after implantation, mice were injected with AAV8-LOXL2 or AAV8-Ctrl via tail vein. As shown in Fig. S7E, expression of LOXL2 in tumor tissues in AAV8-LOXL2 group were upregulated compared to those in AAV8-Ctrl group. Notably, there were no remarkable changes of size and number of orthotopic liver tumors between AAV8-LOXL2 group and AAV8-Ctrl group, however, numbers of lung metastases in AAV8-LOXL2 group were more than those in AAV8-Ctrl group (Fig. S7F-H). Taken together, these findings imply that LOXL2 is involved in RPS7-induced HCC metastasis, probably mostly by promoting FA formation and migration and invasion abilities of HCC cells.

### LOXL2 initiates FA formation by activating ITGB1-mediated FAK/SRC pathway

**Fig. 7:**
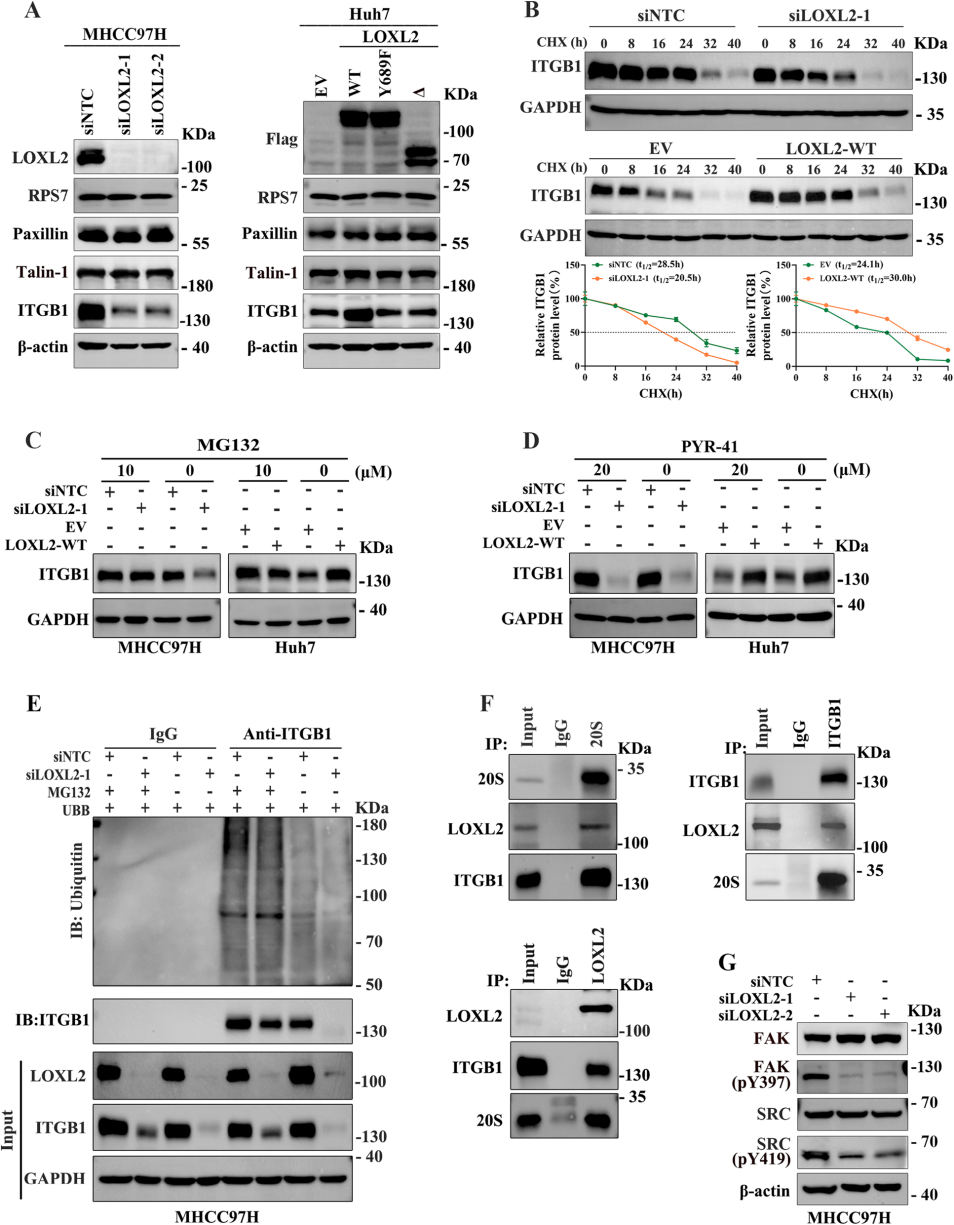
LOXL2 up-regulates ITGB1 via enhancing its protein stability. FA is a well-characterised structure closely regulating tumor cell adhesion and migration, and is consist of numerous proteins. The molecular mechanism underlying the role of LOXL2 in FA formation was further explored. **A** Western blot assay was used to assess the influence of LOXL2 on the expression of several key structure proteins of FA. **B** Protein half-life measurements based on CHX treatment were performed to evaluate the impact of LOXL2 on the stability of ITGB1 protein. **C** Whether LOXL2 regulated ITGB1 protein degradation through the proteasome degradation pathway was determined by western blotting analysis. Cells were treated with 10 μmol/L MG132 for 12 h. **D** Western blotting analysis was performed to determine whether LOXL2 regulated degradation of ITGB1 by proteasome pathway depending on ubiquitin. Cells were treated with 20 μmol/L PYR-41 for 20 h. **E** Whole-cell extracts of LOXL2 knockdown MHCC97H cells were immunoprecipitated with anti-ITGB1 antibody, and ubiquitinated ITGB1 was detected with anti-ubiquitin antibody. **F** A reciprocal co-IP assay was performed in HLE cells. **G** The effect of LOXL2 on signal activity of FAK/SRC pathway in MHCC97H cells was determined by western blotting

FA is a large macromolecular complex that engages with the surrounding extracellular matrix via integrin receptors and physically connects with the actin cytoskeleton through the recruitment of numerous FA-associated proteins, playing a critical role in regulating cell adhesion and motility [17]. To explore the molecular mechanism underlying the role of LOXL2 in FA formation, we firstly investigate the effect of LOXL2 on the expression levels of several essential FA-associated proteins. We found that LOXL2 silencing or overexpressing significantly reduced or upregulated the protein level of ITGB1 respectively, however, had no effect on Talin-1 or Paxillin, another two critical components of FA (Fig. 7A). Taking into account that Paxillin is a cytoskeletal protein involved in actin-membrane attachment at sites of cell adhesion to the extracellular matrix, we performed membrane and cytosolic fractionations as well as western blotting, and found that LOXL2 alterations had no effect on the levels of membrane-associated Paxillin (Fig. S8A), indicating that Paxillin may not be a critical downstream target in the process of LOXL2-mediated FA formation.

Next, using qRT-PCR analysis we found that LOXL2 had no effect on ITGB1 mRNA level (Fig. S8B). Then, the protein half-life measurements based on CHX treatment showed that half-life of ITGB1 protein was remarkably decreased in LOXL2 knockdown cells, while increased after LOXL2 overexpression (Fig. 7B). Our data also showed that the effect of LOXL2 on ITGB1 protein level was blocked in the presence of the proteasome inhibitor MG132 (Fig. 7C). The interaction of LOXL2 and ITGB1 was revealed by a following co-IP assay (Fig. S8C). However, PYR-41 treatment failed to restore ITGB1 protein level regardless of overexpression or knockdown of LOXL2 (Fig. 7D). Consistently, the level of ubiquitinated ITGB1 remained unchanged irrespective of LOXL2 overexpression or knockdown (Fig. 7E and Fig. S8D). These data implied that LOXL2-mediated proteasomal degradation of ITGB1 is not ubiquitin dependent. It is reported that 20 S proteasome complex mainly mediates ubiquitin-independent degradation [18]. Our co-IP assay confirmed the interactions between 20 S, LOXL2 and ITGB1 proteins (Fig. 7F). Moreover, overexpression of LOXL2 weakened the binding of 20 S with ITGB1 (Fig. S8E). These data suggested that LOXL2 could sustain the level of ITGB1 protein via suppressing the ubiquitin-independent proteasome degradation pathway.

Given the fact that FAK and SRC are two crucial integrin-signaling protein in the ITGB1 signaling pathway, we further investigated the effect of LOXL2 on the expression of FAK and SRC, and uncovered that LOXL2 knockdown markedly decreased FAK and SRC phosphorylation without affecting their expression levels in MHCC97H cells (Fig. 7G), implying that LOXL2 activates ITGB1-mediated FAK/SRC signaling pathway.

Furthermore, functional experiments showed that ITGB1 silencing significantly suppressed the enhanced abilities of cell adhesion, migration and invasion abilities as well as FA formation induced by LOXL2 overexpressing (Fig.S9A-E). Importantly, cellular functional experiments revealed that both the FAK inhibitor and SRC inhibitor displayed a significantly reduced adhesion, migration and invasion capacities in Huh7 cells overexpressing LOXL2 (Fig.S10A-C). Moreover, we sought to investigate the function of ITGB1 on RPS7-induced invasive phenotypes of HCC cells. We found that RPS7 knockout markedly decreased ITGB1 protein level and FAK and SRC phosphorylation levels (Fig. S11A). Overexpression of ITGB1 rescued the decreasing abilities of adhesion, migration and invasion of RPS7 knockout MHCC97H cells (Fig. S11B-D). In addition, the FA formation was also apparently decreased in MHCC97H cells with knockout of RPS7, but which could be partially reversed by overexpression of ITGB1(Fig. S11E). Taken together, these findings indicated that LOXL2-mediated activation of ITGB1/FAK/SRC pathway is responsible for RPS7-induced FA formation and signaling transduction, which in turn contributes to RPS7-induced pro-metastasis function in HCC cells.

### Correlation between RPS7 and LOXL2 expression in human HCC

**Fig. 8:**
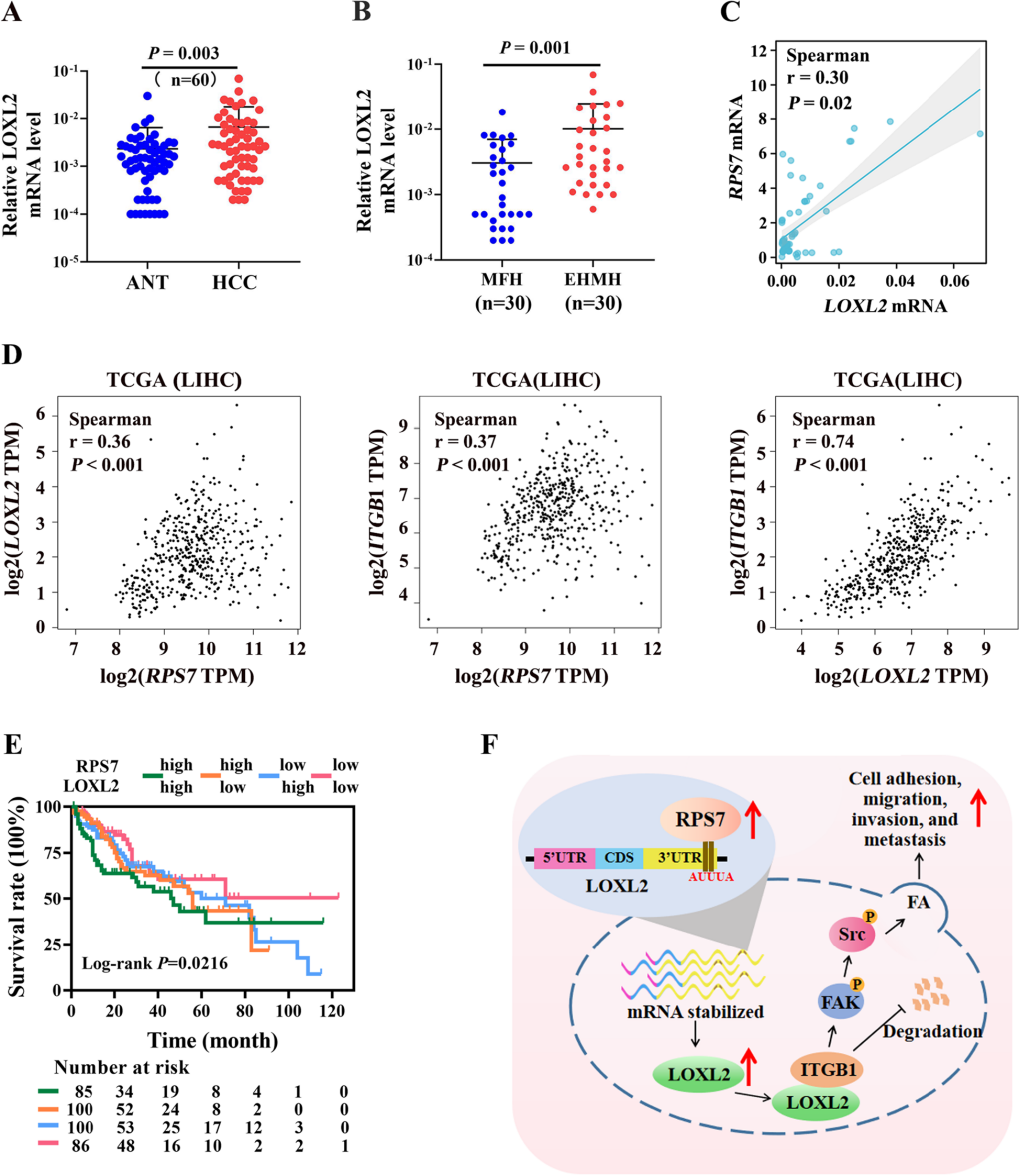
Correlation between RPS7 and LOXL2 expression in human HCC. Sixty paired HCC samples as well as TCGA-LIHC data set were used to evaluate the correlation between RPS7 and LOXL2 expression. **A** The mRNA levels of LOXL2 in HCC tissues compared to matched ANT. **B** The comparison of LOXL2 mRNA levels between HCC with metastasis and metastasis-free HCC. **C** Spearman correlation assay was used to assess the correlation between RPS7 and LOXL2 levels in 60 paired HCC samples. **D** Spearman correlation assay was used to assess the correlation between RPS7, LOXL2 and ITGB1 levels using TCGA-LIHC data set. **E** The impact of RPS7 and LOXL2 expression levels on HCC survival by using TCGA-LIHC data set. **F** The diagram of RPS7 promoting HCC malignant phenotypes via LOXL2-mediated focal adhesion signaling

To elucidate the correlation between RPS7 and LOXL2 expression in human HCC, we furtherly analyzed the mRNA level of LOXL2 in our 60 HCC samples and uncovered that mRNA levels of LOXL2 in HCC tissues were remarkably higher than those in normal liver tissues (Fig. 8A). We also found that the mRNA levels of LOXL2 in HCC tissues with metastasis were significantly higher than those in metastasis-free HCC tissues (Fig. 8B). According to Spearman correlation analysis, we found that there was a significant positive correlation between RPS7 and LOXL2 mRNA level in HCC (Fig. 8C). Through our analysis of public data sets in the TCGA-LIHC database, we further confirmed the positive correlation between RPS7, LOXL2 and ITGB1 mRNA levels in HCC tissues (Fig. 8D). Importantly, we revealed that HCC patients with low RPS7 expression and concomitantly low LOXL2 expression had a longer overall survival than those in control groups (Fig. 8E). Collectively, these results suggested that RPS7-LOXL2-ITGB1 axis may play an important role in HCC progression.

## Discussion

Metastasis is the leading cause of cancer-associated deaths and is particularly salient in HCC [19]. However, the precise mechanisms of involvement in HCC metastasis are still largely unknown, as reflected in the dearth of molecular markers associated with HCC progression and metastasis [20]. Given the essential role of RBPs in many cellular activities, their extensive involvement in cancer is unsurprising. In recent years, RBP dysregulation has been demonstrated to be linked to a variety of cancers, including HCC [21]. Nevertheless, to date, only a few HCC-related RBPs have been well characterized. In the present study, based on our previous RNA-seq data originating from primary HCC tissues with or without extrahepatic metastasis, we identified a total of 362 differentially expressed RBPs in EHMH compared to MFH tissues, indicating that the aberrant RBPs might also closely participate in the metastasis of HCC.

It is well-known that the basic function of RPs is to participate in the maintenance of specialized structure stability of ribosomal RNA (rRNA), thereby assisting in correct rRNA folding. However, with the further structural and function studies of RPs, it was found that the regulatory role of RPs family on RNA may have been grossly underestimated. More and more studies have reported that RPs could regulate post-transcriptional steps of gene expression, including pre-mRNA splicing, and mRNA transport, stability and translation, through binding to mRNA, thus involve in the regulation of some cellular processes including proliferation, apoptosis, angiogenesis, invasion, and metastasis [8, 22, 23]. Several aberrantly expressed RPs have been reported in HCC. RPL28 is considered a key gene that induces sorafenib resistance in HCC [24]. Grinchuk and colleagues developed a 24-ribosomal gene-based HCC classifier which could be introduced as a robust and reproducible prognostic model for stratifying HCC patient risks [25], suggesting a promising practical application in clinic. Nevertheless, until now, many HCC progression-related RPs remain to be discovered. In the current study, for the first time, we identified RPS7, a ribosomal protein that constitutes the 40 S ribosomal subunit, as a potential novel predictor of prognosis for HCC patients. We found that the level of RPS7 was significantly upregulated in HCC tissues, and was particularly upregulated in HCC tissues with metastasis. We also found that high level of RPS7 was strongly positively correlated with a poor clinical outcome of HCC patients. Altogether, our data indicated that RPS7 has a potential to become a valuable biomarker for patients with HCC.

Previous studies have demonstrated that RPS7 mutation is strongly correlated with Diamond-Blackfan Anemia [26, 27]; however, more and more researches have recently focused on the effect of RPS7 in tumors. In terms of liver cancer, Danele et al. reported that RPS7 was transcriptionally regulated by c-Myc and might play a role in c-Myc-mediated hepatocellular malignant transformation [28], but the exact role of RPS7 in the regulation of HCC cell function has not been elucidated. In this study, we demonstrated the pro-proliferation, pro-adhesion, pro-invasion and pro-metastasis effects of RPS7 in HCC cells according to in vitro and in vivo studies. Importantly, for the first time, we identified LOXL2 was as an important downstream target of RPS7, and demonstrated that LOXL2 was essential for sustaining RPS7-induced HCC cells invasion and metastasis. Given the potential RNA binding ability of RPS7, we confirmed the binding of RPS7 protein to LOXL2 mRNA, as well as the specific binding sites within LOXL2 mRNA, by RNA RIP and RNA pull-down assays. It is well known that RNA binding domains are critical elements and functional vectors of RBPs. Several conserved motifs and domains including the RNA recognition motifs (RRMs), the K homology (KH) domain, zinc fingers (ZnFs), and Pumilio homology domain (PUM-HD), have been revealed in some RBPs [6]. Related studies have revealed RNA binding domains in several RPs including RPL1, RPL11 and RPS6 [29–31]; nevertheless, the RNA binding domain of RPS7 has not been elucidated. Whether RPS7 containing one or more such domains needs to be further explored, which is also the part we will continue to study in depth in the follow-up work.

FA is a well-characterised structure closely regulating cell adhesion and migration. Increasing researches have revealed its critical roles in HCC cells invasion and metastasis [32, 33]. The formation and maturation of FA is modulated by several extracellular and intracellular mechanisms. LOXL2 is an enzyme which stabilizes collagen crosslinking, whose enzyme activity has been reported to be closely correlated with a variety of diseases, such as cardiac fibrosis, Uterine hypertrophy and cancer [34–36]. In addition to its catalytic role, the pro-oncogenic effect of LOXL2 on some cancers is attributed to its intracellular functions, such as transcriptional regulation of EMT-related genes expression [37], and a scaffolding protein role [38]. A previous study has uncovered the effect of extracellular LOXL2 on FA formation and maturation [39]. However, the role and mechanism of intracellular LOXL2 protein on FA formation has not been elucidated yet. Some representative biological events including ITGB1 activation and concomitant activation of several signaling molecules such as FAK and SRC were recognized as hallmarks of FA formation and maturation, and have also been demonstrated to be particularly important in cancer metastasis. Our data revealed that LOXL2 could up-regulate ITGB1 protein level and activate ITGB1-mediated signaling pathway, which is congruous with other reports in the literature [36, 40]. As a membrane protein, ITGB1 has been reported to be ubiquitinated, internalized, and degraded by the lysosome [41]. Moreover, it was found that intracellular ITGB1 could be degraded by proteasome [42]. The effect of LOXL2 on upregulating ITGB1 expression via inhibiting degradation of ITGB1 protein by proteasome pathway has been reported in clear cell renal cell carcinoma [43], however the authors did not do any further studies to investigate the interactions of the two proteins. Our data uncovered that LOXL2-mediated degradation of ITGB1 protein by proteasome pathway does not depend on ubiquitination, and further confirmed the interaction of LOXL2 with ITGB1 protein and 20 S through a reciprocal co-IP assay. These results yield a novel insight into the role of LOXL2 upregulating ITGB1 expression in human cancer cells.

## Conclusions

In conclusion, we report RPS7 as a novel oncogenic RBP in HCC that contributes to HCC metastasis by posttranscriptionally regulating the expression of LOXL2 and activating LOXL2-mediated ITGB1/FAK/SRC signaling pathway (Fig. 8F). Our data reveal the critical roles of the RPS7/LOXL2/ITGB1 axis in HCC metastasis and shed new light on the exploration of molecular drugs against HCC.

### Supplementary Information

**Additional file 1: Supplementary materials and methods. Supplementary Table 1.** Primers of different LOXL2 related fragments. **Supplementary Table 2.** Primers of qRT-PCR in this study**. Supplementary Table 3.** Primers of RNA pull-down in this study**. Supplementary Table 4.** Primers of dual luciferase reporter assay in this study**. Supplementary Fig 1.** Identification of RPS7 as an important gene closely associated with HCC progression. A. The correlation between expression levels of each gene and overall survival rate in HCC with metastases. B. The differential expression of RPS7 in other frequent cancers except HCC. C. The correlation between RPS7 expression and HCC histological grades and clinical stages, respectively. ***, *P* < 0.001. **Supplementary Fig 2.** The expression of RPS7 in HCC tissues and matched normal liver tissues. Western blot was performed to detect RPS7 protein levels in EHMH group and MFH group, respectively. GAPDH was used as loading control. EHMH, HCC tissues with extrahepatic metastasis; MFH, metastasis-free HCC tissues.** Supplementary Fig 3.** Effect of RPS7 on HCC cell phenotypes in vitro. A and B. Effect of PRS7 knockout on MHCC97H and HLE proliferation was determined by CCK-8 assay (A) and colony formation assays (B). C and D. Effect of RPS7 overexpression on Huh7 and PLC/PRF/5 cells proliferation was determined by CCK-8 assay (C) and colony formation assays (D). E. The wound closure abilities of RPS7-knockout cells were determined by wound healing assay. Representative data are from at least 3 independent experiments. Data are shown as mean ± SD. **, *P* < 0.01.** Supplementary Fig 4.** Overexpression of RPS7 promotes HCC cell adhesion, migration and invasion in vitro and metastasis in vivo. Huh7 and PLC/PRF/5 cells, two poorly aggressive HCC cell lines, were used to establish stable RPS7 overexpression cells via lentivirus carrying RPS7 (Lv-RPS7). Cells infected with lentivirus carrying empty vector were correspondingly used as controls (Lv-EV). The cell-matrix adhesion capacity, migration and invasion ability of cells in vitro as well as metastasis in vivo were observed under RPS7 overexpression. A. Overexpression efficiency was determined by western blot assay. B. RPS7-overexpressed HCC cells adhesion to fibronectin, collagen I and collagen IV were detected using cell-matrix adhesion assay. C. The wound closure abilities of RPS7-overexpressed cells were determined by wound healing assay. D. The effect of RPS7-overexpressed on cell migration and invasion were determined by Transwell assay. E. A orthotopic mouse model was constructed using RPS7-overexpressed Huh7 cells and control cells (each group, *n*=12). The effect of RPS7-overexpressed on tumor size and numbers were evaluated. F and G. The effects of RPS7-overexpressed on lung metastasis were evaluated by orthotopic mouse models and tail vein lung metastasis mouse models, respectively. Representative data are from at least 3 independent experiments. Data are shown as mean ± SD. *, *P*< 0.05, **, *P* < 0.01.** Supplementary Fig 5.** Effect of RPS7 on LOXL2 expression at transcriptional level. A. Effect of RPS7 overexpressing/knockdown on LOXL2 promoter activity was detected using dual luciferase reporter assay, respectively. B. Nascent LOXL2 RNA was measured by qRT-PCR following pull-down of biotin-conjugated, EU-labeled RNA in RPS7 knockdown or overexpressed HCC cells. Representative data are from at least 3 independent experiments. Data are shown as mean ± SD. ns, no significant.** Supplementary Fig 6.** LOXL2 mediates RPS7-induced cell adhesion, migration and invasion. A. Catalytic activity deletion mutant of LOXL2 (LOXL2-Δ) and catalytically inactive point mutant of LOXL2 (LOXL2-Y689F), as well as wild type LOXL2 (LOXL2-WT) were respectively constructed. B. Such plasmids were transfected into Huh7 cells. Effect of LOXL2 overexpression was determined by western blotting. C. Effect of LOXL2 silencing was determined by western blotting. D. The impact of 20 μM CMMH on LOXL2 enzyme activity was evaluated using a lysyl oxidase assay kit. MHCC97H cells were treated with 20 μM CMMH, a highly selective LOXL2 enzyme inhibitor, the effect of CMMH on cell-matrix adhesion ability (E) and migration and invasion (F) were analyzed. G. Cell-matrix adhesion assay was performed to evaluate the effect of 20 μM CMMH on the adhesion ability of RPS7-overexpressed Huh7 cells. H. Transwell assay was performed to assess the effect of 20 μM CMMH on the migration and invasion abilities of RPS7-overexpressed Huh7 cells. I. Cell-matrix adhesion assay was performed to evaluate the effect of LOXL2 overexpressing on the adhesion ability of RPS7-knockout MHCC97H cells. J. Transwell assay was performed to assess the effect of LOXL2 overexpressing on the migration and invasion abilities of RPS7-knockout MHCC97H cells. Data are shown as mean ± SD. **, *P* < 0.01. ns, no significant.** Supplementary Fig 7.** LOXL2 is involved in RPS7-mediated HCC metastasis in vivo. A-D. RPS7-overexpressed Huh7 cells were orthotopically injected into the left lobe of orthotopic liver of the nude mice. Four weeks after inoculation, CMMH (15 mg/kg) was administered twice a week for six weeks through tail vein injection. Expression of LOXL2 and RPS7 in tumor tissues were detected by western blot (A). Tumor size and numbers were evaluated in CMMH-treated mice compared to control group (B). Lung metastasis were evaluated according to detect metastatic nodules and foci (C and D). E-H. Orthotopic HCC models were established by using RPS7-deletion MHCC97H cells. At 2 weeks after implantation, mice were injected with AAV8-LOXL2 (1 × 10^11^ viral genomes in 100 μL saline) or AAV8-Ctrl (1 × 10^11^ viral genomes in 100 μL saline) via tail vein. Expression of LOXL2 and RPS7 in tumor tissues were detected by western blot (E). Tumor size and numbers were evaluated between each group (F). Lung metastasis were evaluated according to detect metastatic nodules and foci (G and H). Representative data are from at least 3 independent experiments. Data are shown as mean ± SD. **, *P* < 0.01. ns, no significant.** Supplementary Fig 8.** LOXL2 regulates ITGB1 expression at protein level. A. Membrane and cytosolic fractionations was performed to explore whether LOXL2 alterations affects the levels of membrane-associated Paxillin. B. qRT-PCR was performed to detect the influence of LOXL2 on ITGB1 mRNA level. C. Co-immunoprecipitation (co-IP) assay was performed in HLE cells to evaluate the interact between LOXL2 and ITGB1. D. Whole-cell extracts of LOXL2 overexpressed PLC/PRF/5 cells were immunoprecipitated with anti-ITGB1 antibody, and ubiquitinated ITGB1 was detected with anti-ubiquitin antibody. E. The immunoprecipitation of 20S and ITGB1 was detected in PLC/PRF/5 cells transfected with empty vector or LOXL2-WT plasmids. Data are shown as mean ± SD. ns, no significant.** Supplementary Fig 9. **ITGB1 is involved in LOXL2-mediated malignant phenotypes of HCC cells. A. The effect of ITGB1 silencing in Huh7 cells was determined by western blotting. B-E. Functional cell experiments were performed to evaluate the effect of ITGB1 on LOXL2-overexpressed Huh7 cells adhesion capacity (B), wound closure ability (C), migration and invasion abilities (D) and focal adhesion formation (E). Representative data are from at least 3 independent experiments. Data are shown as mean ± SD. **, *P* < 0.01. ns, no significant. **Supplementary Fig 10. **FAK/SRC signaling is involved in LOXL2-mediated invasive phenotype of HCC cells. LOXL2-overexpressed Huh7 cells were treated with Defactinib, a FAK inhibitor and PP2, a SRC inhibitor, for 48h, respectively. Functional cell experiments were performed to evaluate the effect of such inhibitors on cells adhesion capacity (A), wound closure ability (B), migration and invasion abilities (C). Representative data are from at least 3 independent experiments. Data are shown as mean ± SD. **, *P* < 0.01. ns, no significant.** Supplementary Fig 11.**ITGB1 is involved in RPS7-mediated invasive phenotype of HCC cells. A. The effect of RPS7 knockout on signal activity of ITGB1/FAK/SRC pathway was determined by western blotting. B-E. Functional cell experiments were performed to evaluate the effect of ITGB1 on RPS7-knockout MHCC97H cells adhesion capacity (B), wound closure ability (C), migration and invasion abilities (D) and focal adhesion formation (E). Representative data are from at least 3 independent experiments. Data are shown as mean ± SD. *, *P* < 0.05, **, *P* < 0.01.

## Data Availability

The datasets generated and analyzed during the current study are available from the corresponding author on reasonable request.

## Abbreviations

HCC: Hepatocellular carcinoma
RPs: Ribosomal proteins
LOXL2: Lysyl oxidase-like 2
EHMH: Primary HCC tissues with extrahepatic metastasis
MFH: Metastasis-free HCC tissue
RBPs: RNA-binding proteins
RPS7: Ribosomal protein S7
RNA-seq: RNA sequencing
TCGA-LIHC: The Cancer Genome Atlas–Liver Hepatocellular Carcinoma
DERBPs: Differentially expressed RBPs
