# Correction: RNA-binding protein RPS7 promotes hepatocellular carcinoma progression via LOXL2-dependent activation of ITGB1/FAK/SRC signaling

**DOI:** 10.1186/s13046-024-02999-9

**Published:** 2024-03-14

**Authors:** Yu-Jiao Zhou, Min-Li Yang, Xin He, Hui-Ying Gu, Ji-Hua Ren, Sheng-Tao Cheng, Zhou Fu, Zhen-Zhen Zhang, Juan Chen

**Affiliations:** 1 Department of Infectious Disease, Children’s Hospital of Chongqing Medical University, National Clinical Research Center for Child Health and Disorders, Ministry of Education Key Laboratory of Child Development and Disorders, Chongqing Key Laboratory of Child Rare Diseases in Infection and Immunity, No.20 Jinyu Road, Chongqing, 401122 Yubei District China; 2 The Key Labora- Tory of Molecular Biology of Infectious Diseases Designated By the Chinese Ministry of Education, Chongqing Medical University, Chongqing, China; 3 Stem Cell Biology and Therapy Laboratory, Ministry of Education Key Labora- Tory of Child Development and Disorders, and the Department of Respiratory Diseases, The Children’s Hospital of Chongqing Medical University, Chongqing, China; 4 Key Laboratory of Laboratory Medical Diagnostics, Chinese Ministry of Education, Chongqing Medical University, No.1 Youyi Road, Chongqing, 400016 Yuzhong District China

**Correction: J Exp Clin Cancer Res 43, 45 (2024)**

**https://doi.org/10.1186/s13046-023-02929-1**

Following publication of the original article [1], errors were spotted particularly in Fig. 6, specifically:

Fig. 6b – upper panels, incorrect representative image used for migration transwell assays of Huh7 cells transfected with EV

Fig. 6d – lower panels, incorrect representative image used for invasion transwell assays of MHCC97H cells transfected with siNTC

Authors missed the Production Editor’s email which resulted to not being able to check the modification status of Fig. 6. The correct figure is given below. The correction does not affect the overall result or conclusion of the article. The original article has been corrected.

Incorrect Fig. 6

**Fig. 6:**
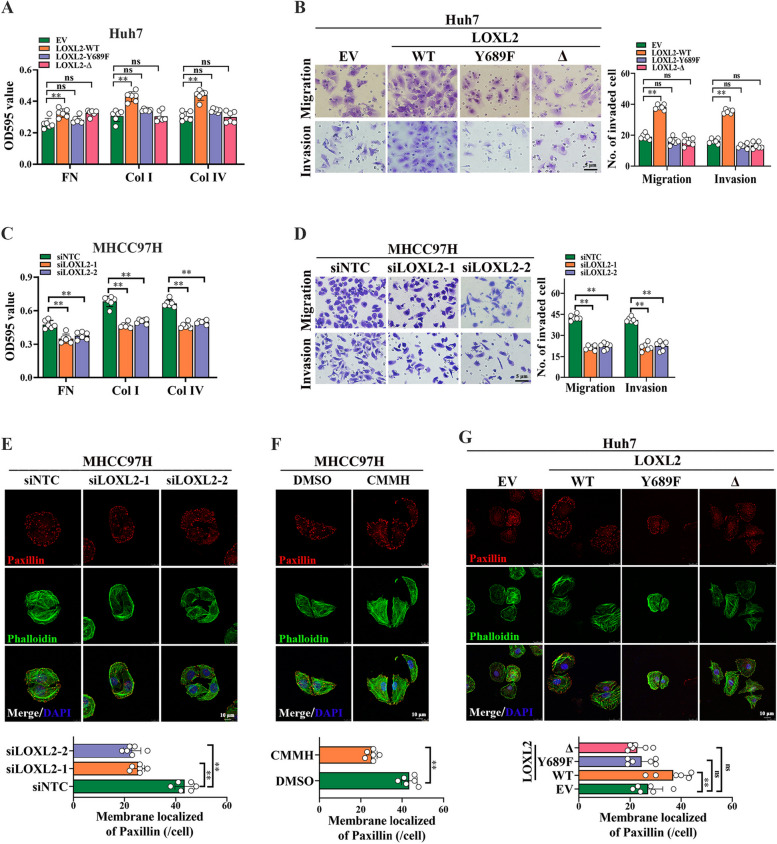
LOXL2 promotes focal adhesion (FA) formation, migration and invasion of HCC cells. **A** and **B** To evaluate the role of LOXL2 in HCC metastasis, two pcDNA3.1-LOXL2-3 × Flag mutant plasmids, catalytic activity deletion mutant of LOXL2 (LOXL2-Δ) and catalytically inactive point mutant of LOXL2 (LOXL2-Y689F), as well as wild type LOXL2 (LOXL2-WT) were respectively constructed and stably transfected into Huh7 cells. The effect of LOXL2 overexpression on cell–matrix adhesion ability (**A**) and migration and invasion (**B**) were analyzed. **C** The effect of LOXL2 silencing on cell–matrix adhesion ability in MHCC97H cells. **D** The effect of LOXL2 silencing on cell migration and invasion in MHCC97H cells. **E–G**. The effect of LOXL2 silencing (**E**), 20 μM CMMH (**F**), and LOXL2 overexpression (**G**) on FA formation in HCC cells were respectively detected by Immunofluorescence staining experiments. Representative data are from at least 3 independent experiments. Data are shown as mean ± SD. **, P < 0.01. ns, no significant

Correct Fig. 6

**Fig. 6:**
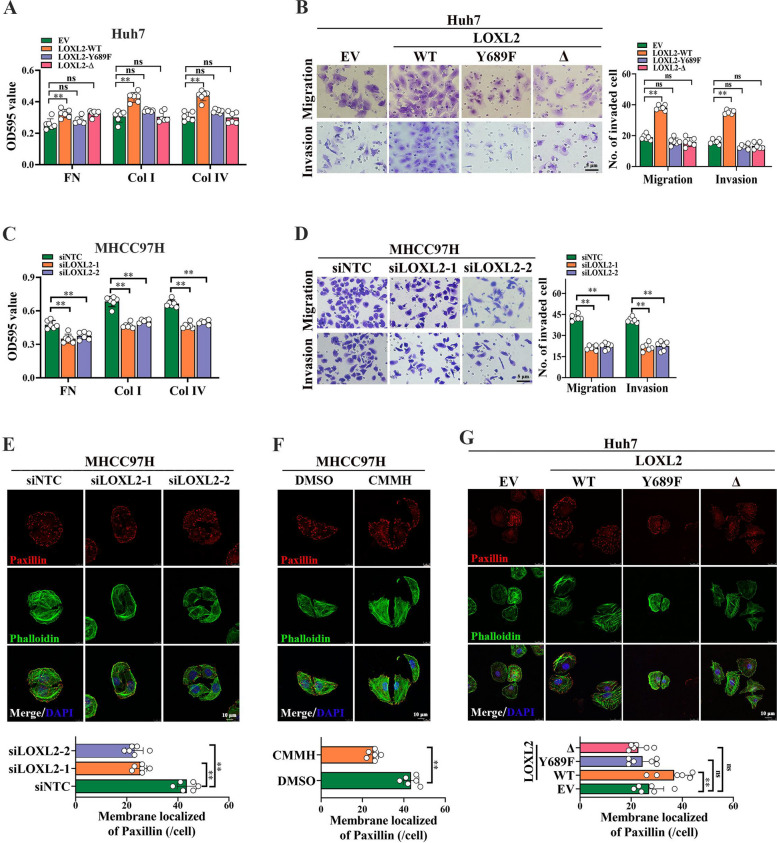
LOXL2 promotes focal adhesion (FA) formation, migration and invasion of HCC cells. **A** and **B** To evaluate the role of LOXL2 in HCC metastasis, two pcDNA3.1-LOXL2-3 × Flag mutant plasmids, catalytic activity deletion mutant of LOXL2 (LOXL2-Δ) and catalytically inactive point mutant of LOXL2 (LOXL2-Y689F), as well as wild type LOXL2 (LOXL2-WT) were respectively constructed and stably transfected into Huh7 cells. The effect of LOXL2 overexpression on cell–matrix adhesion ability (**A**) and migration and invasion (**B**) were analyzed. **C** The effect of LOXL2 silencing on cell–matrix adhesion ability in MHCC97H cells. **D** The effect of LOXL2 silencing on cell migration and invasion in MHCC97H cells. **E–G**. The effect of LOXL2 silencing (**E**), 20 μM CMMH (**F**), and LOXL2 overexpression (**G**) on FA formation in HCC cells were respectively detected by Immunofluorescence staining experiments. Representative data are from at least 3 independent experiments. Data are shown as mean ± SD. **, P < 0.01. ns, no significant
