# Supplementary material for: RNA-binding protein RPS7 promotes hepatocellular carcinoma progression via LOXL2-dependent activation of ITGB1/FAK/SRC signaling
Source: J Exp Clin Cancer Res. 2024 Feb 8;43:45. doi: 10.1186/s13046-023-02929-1 (PMC10851485; doi:10.1186/s13046-023-02929-1)
Supplement: Supplementary file 1 — Additional file 1: Supplementary materials and methods. Supplementary Table 1. Primers of different LOXL2 related fragments. Supplementary Table 2. Primers of qRT-PCR in this study. Supplementary Table 3. Primers of RNA pull-down in this study. Supplementary Table 4. Primers of dual luciferase reporter assay in this study. Supplementary Fig 1. Identification of RPS7 as an important gene closely associated with HCC progression. A. The correlation between expression levels of each gene and overall survival rate in HCC with metastases. B. The differential expression of RPS7 in other frequent cancers except HCC. C. The correlation between RPS7 expression and HCC histological grades and clinical stages, respectively. ***, P < 0.001. Supplementary Fig 2. The expression of RPS7 in HCC tissues and matched normal liver tissues. Western blot was performed to detect RPS7 protein levels in EHMH group and MFH group, respectively. GAPDH was used as loading control. EHMH, HCC tissues with extrahepatic metastasis; MFH, metastasis-free HCC tissues. Supplementary Fig 3. Effect of RPS7 on HCC cell phenotypes in vitro. A and B. Effect of PRS7 knockout on MHCC97H and HLE proliferation was determined by CCK-8 assay (A) and colony formation assays (B). C and D. Effect of RPS7 overexpression on Huh7 and PLC/PRF/5 cells proliferation was determined by CCK-8 assay (C) and colony formation assays (D). E. The wound closure abilities of RPS7-knockout cells were determined by wound healing assay. Representative data are from at least 3 independent experiments. Data are shown as mean ± SD. **, P < 0.01. Supplementary Fig 4. Overexpression of RPS7 promotes HCC cell adhesion, migration and invasion in vitro and metastasis in vivo. Huh7 and PLC/PRF/5 cells, two poorly aggressive HCC cell lines, were used to establish stable RPS7 overexpression cells via lentivirus carrying RPS7 (Lv-RPS7). Cells infected with lentivirus carrying empty vector were correspondingly used as controls (Lv-EV). The cell-mat [file 13046_2023_2929_MOESM1_ESM.docx]

**Supplementary information**

Table of contents

Supplementary materials and methods ................................................................... 2

Supplementary Table 1............................................................................................10

Supplementary Table 2............................................................................................11

Supplementary Table 3............................................................................................12

Supplementary Table 4............................................................................................13

Supplementary Fig.1...............................................................................................14

Supplementary Fig.2...............................................................................................15

Supplementary Fig.3...............................................................................................16

Supplementary Fig.4...............................................................................................17

Supplementary Fig.5...............................................................................................19

Supplementary Fig.6...............................................................................................20

Supplementary Fig.7...............................................................................................22

Supplementary Fig.8...............................................................................................24

Supplementary Fig.9...............................................................................................25

Supplementary Fig.10.............................................................................................26

Supplementary Fig.11.............................................................................................27

**Supplementary materials and methods**

**Clinical samples**

Sixty paired human HCC liver tissues and matched nontumoral liver tissues (metastasis *vs* non-metastasis = 30 *vs* 30) were collected from patients at the First Affiliated Hospital of Chongqing Medical University, Chongqing, China. Written informed consent was obtained3 from the patients. Tissue specimens collected after surgical resection were flash-frozen in liquid nitrogen and stored at −80 °C for further investigation. The study protocol conformed to the 1975 Declaration of Helsinki ethical guidelines and was approved by the Ethics Committee of Chongqing Medical University.

**Lentivirus, plasmids and small interfering RNA (siRNA)**

For RPS7 overexpression, full-length cDNA encoding human RPS7 (NM_001011.4) was cloned into the lentiviral vector (CMV-MCS-3FLAG-EF1-ZsGreen1-T2A-puromycin) and used to infect the indicated cell lines. Lentiviruses were purchased from the Genechem Company (Shanghai, China), and lentivirus infection was performed according to the instructions. Overexpression/knockdown efficiency was examined by western blot assay and qRT-PCR assay.

Plasmids (pcDNA3.1-3×FLAG backbone) containing the wild type LOXL2 (LOXL2-WT), deletion mutants of LOXL2 (LOXL2-Δ: deleted aa 548-774), and a point mutant of LOXL2 (LOXL2-Y689F) were constructed. The siRNAs targeting the indicated genes were purchased from GenePharma company. The primer sequences are listed in Table S1. The indicated cell lines were transfected with the aforementioned plasmids or siRNAs with the use of Lipofectamine 3000 reagent, and the overexpression or silencing efficiency was examined by western blot assay.

**CRISPR/Cas9-mediated gene knockout**

CRISPR/Cas9-mediated gene editing was performed to establish RPS7-knockout MHCC97H cells. In brief, an efficient small guide RNA (sgRNA) sequence targeting RPS7 (sense: 5′-CACCGCCAAGATCGTGAAGCCCAA-3′) and a human nontargeting sgRNA (sense: 5′-ACGGAGGCTAAGCGTCGCAA-3′) that does not recognize any sequence were designed using online tools (http://www.e-crisp.org/E-CRISP/). These sgRNAs were inserted into the LentiCRISPR V2 vector. Then, 293T cells were cotransfected with vectors containing sgRNA (lentiCRISPR-V2-sgRNA), lentiviral packaging plasmid psPAX2 and lentivirus envelope vector pMD2. G (lentiCRISPR-V2-sgRNA: psPAX2: pMD2.G=4:3:1). The supernatants were harvested after transfection for 48 hours and then utilized for infection with MHCC97H cells. After selection with puromycin (2 μg/mL) for 2 weeks, a single cell clone was isolated. “TA” cloning was used to identify the knockout alleles, and a western blot assay was performed to further determine the knockout efficiency.

**RNA-seq analysis**

This study focused on the differentially expressed RBP-related genes based on RNA-seq analysis. Specifically, we first obtained the logFC value of all the transcripts using edgeR computing. Then, the limma software package was used to calculate the differentially expressed genes (DEGs) in each group. Briefly, DEGs between EHMH and ANT, MFH and ANT, and EHMH and FMH tissues were analyzed using the DEseq2 software package. The cutoff criteria were P value < 0.05 and | log2FC | ≥ 1. Then, the differentially expressed RBP (DERBP) genes were selected from the above three gene sets. Ultimately, the shared DERBPs were identified by overlapping these three gene sets.

With respect to cells, RPS7-knockdown MHCC97H cells and negative control cells were subjected to RNA-seq analysis. Total RNA was isolated using an RNeasy mini kit (Tiangen, 74106). Paired-end libraries were synthesized by using the TruSeq™ RNA Sample Preparation Kit (Illumina, RS-930-2002) following the TruSeq™ RNA Sample Preparation Guide. Briefly, the poly-A-containing mRNA molecules were purified using poly-T oligo-attached magnetic beads. Library construction and sequencing were performed by Sinotech Genomics Co., Ltd. (Shanghai, China). Gene abundance was expressed as fragments per kilobase of exon per million reads mapped (FPKM). StringTie software was used to count the fragments within each gene, and the TMM algorithm was used for normalization. Differential expression analysis for mRNA was performed using the R package edgeR. Differentially expressed RNAs with |log2(FC)| value ≥1 and P value <0.05, considered significantly modulated, were retained for further analysis.

**Quantitative real-time PCR (qRT–PCR) analysis**

Total RNA was extracted from cultured cell lines and HCC tissues using TRIzol reagent (Tiangen, DP424) and an RNA isolation kit (Beyotime, R0026), respectively. cDNA was synthesized using the FastKing RT kit (Tiangen, KR116). Then, the mRNA levels of target genes were detected by qRT–PCR with SYBR® Green Supermix (BioRad, 1725120). The primer sequences are listed in Table S2. The relative mRNA levels of target genes were calculated by the 2^−ΔΔCt method.

**Western blot analysis**

Briefly, total proteins were lysed using RIPA lysis buffer with protease inhibitor cocktails (Roche, 4693132001). BCA protein assay reagent (Thermo Scientific, 23225) was used to detect the protein concentrations. The designated protein lysates were separated via SDS–PAGE and then transferred onto PVDF membranes. After blocking with 5% nonfat milk (blocking solution) for 2 h at room temperature, membranes were incubated with primary antibodies against RPS7 (Novus, NBP1-57394), LOXL2 (Proteintech, 67139-1-Ig), ITGB1 (Proteintech, 12594-1-AP), Talin-1 (Proteintech,14168-1-AP), Paxillin (Abcam, [ab32084)](https://www.abcam.cn/products/primary-antibodies/paxillin-antibody-y113-ab32084.html)), FAK (Zenbio, R24276), p-FAK (Zenbio, 381143), SRC (Santa Cruz, sc-130124), p-SRC (Santa Cruz, sc-81521), 20S (Proteintech, 11943-2-AP) or GAPDH (Bioworld, BS72410) overnight at 4 °C on a shaker. After incubation with a secondary antibody, the signals were visualized by ECL (Millipore, WBKLS0500).

**Immunohistochemistry**

Tissue samples were fixed with formalin and embedded in paraffin. In brief, tissue sections were sequentially deparaffinized, dehydrated, and antigen retrieved and then incubated with 3% hydrogen peroxide to block endogenous peroxidase. Following blocking with 10% normal goat serum for 1 h at room temperature, the sections were incubated with primary antibodies at 4 °C overnight and HRP-conjugated secondary antibody for 30 min at room temperature. Finally, immunoreactivity was detected using 3,3-diaminobenzidine (DAB), followed by restaining with hematoxylin. Images were captured under a microscope.

**Cell proliferation detection and colony formation assay**

The effect of RPS7 overexpression or knockdown/knockout on cell proliferation was measured by CCK8 assay. Briefly, cells were seeded into 96-well plates in triplicate at a density of 2000 cells per well. Cell viability was measured at the indicated time points using the Cell Counting Kit (MCE, HY-K0301) according to the manufacturer’s instructions.

In addition, the effect of RPS7 on colony-forming ability was determined by a colony formation assay. Briefly, the indicated cells were seeded into 6-well plates at a density of 1000-1500 cells per well and cultivated for 10-12 days. The colonies were fixed with methanol and stained with crystal violet for 20 min. Each experiment was repeated at least three times.

**Cell-matrix adhesion assay**

The indicated cells were plated at a density of 5000 cells per well in 96-well plates coated with fibronectin, collagen I or collagen IV (10 μg/ml). The cells were incubated at 37 °C for 2 h. After washing three times with PBS, the attached cells were fixed with 1% glutaraldehyde for 30 min and stained with 0.1% crystal violet in PBS for 30 min. After washing three times with PBS, the stained cells were homogenized with 10% acetic acid for 10 min. The absorbance of each well of the plates was measured at 595 nm.

**Wound-healing assay**

The indicated cells were seeded into 6-well plates and grown to 90% confluence the following day. Before wound creation, the cells were treated with 5 µg/mL mitomycin-C (MCE, HY-13316) for 2 hours. After washing with PBS three times, the wells were refilled with 2 mL growth medium. Images of scratches were captured at 0 h and 48 h under an inverted microscope. The migrated rate (%) was calculated using the following formula: Relative gap area = (gap area at X h/total area) × 100%.

**Transwell migration and invasion assay**

HCC cells were pretreated in the presence of 5 µg/mL mitomycin-C for 2 hours, and the indicated amount of cells was resuspended in 500 μL of serum-free medium and seeded into a Transwell chamber coated with (invasion assay) or without (migration assay) Matrigel (BD Biosciences, 356234). Cells were incubated at 37 °C for 12 h for the migration assay and 24 h for the invasion assay. After fixation with methanol, the inside cells were scraped with a cotton swab, and the underside cells were stained with 0.1% crystal violet. The migrated or invaded cells (6 fields per chamber) were counted under an inverted microscope.

**RNA immunoprecipitation (RIP) assay**

MHCC97H cells were used to perform RIP experiments using an anti-RPS7 antibody (Santa Cruz, sc-377317) or isotype-matched IgG antibody (Millipore, AP101). Following the recovery of antibodies using protein A/G beads, qRT–PCR was performed on the precipitates to detect LOXL2 and GAPDH mRNA levels. The primer sequences are shown in Table S2.

**RNA pull-down assay**

The main workflows of this experiment were consistent with those described in the manufacturer’s protocol. Here, a brief summary is provided. cDNA was used as a template for PCR amplification of the different fragments of LOXL2 mRNA, such as the 5’UTR, CDS, 3’UTR, and fragments within the 3’UTR, including 2576-2975 nt, 2976-3375 nt, 3376-3721 nt, 2976-3175 nt, and 3155-3375 nt, as well as the wild-type and three different mutants within 3190-3259 nt. The primer sequences are listed in Table S3. Then, the amplified product was transcribed in vitro by T7 RNA polymerase by using the AmpliScribe^TM^ T7 High Yield Transcription Kit (Ribo, C11002). Transcribed RNAs were subsequently biotin-labeled with the Biotin RNA Labeling Mix (Thermo Scientific, 20163). Next, 100 pmol biotinylated RNA was chemically coupled to streptavidin-linked magnetic beads (75 μL) at room temperature for 2 h (Thermo Scientific, 20164). Two hundred micrograms of total protein extracted from MHCC97H cells was added to the RNA-loaded beads and incubated overnight at 4℃. After washing 3 times, precipitated proteins were eluted in 50 μL protein lysis buffer. Following separation by SDS–PAGE, the pull-down materials were subsequently analyzed by western blotting with RPS7-specific and GAPDH-specific antibodies.

**Dual luciferase assay**

LOXL2 transcriptional activity was determined using a dual-luciferase reporter assay system. The primer pairs used for the construction of pGL3-derived reporter vectors bearing the LOXL2 promoter with the Mlul or Xhol restriction enzyme cutting site are listed in Table S4. For reporter gene assays, the constructed luciferase reporter vectors and Renilla vectors as loading controls were cotransfected using Lipofectamine 3000 reagent (Thermo Scientific, L3000001) following the manufacturer’s instructions. After incubation for 48 h, the supernatant of the cell lysates was collected, and luciferase activity was measured using the Dual-Luciferase Reporter Assay System (Promega, E1910) and normalized to Renilla luciferase activity.

Additionally, to confirm the binding interaction between RPS7 and LOXL2 AUUUA motifs, a pmirGLO-derived luciferase reporter containing wild-type and three different mutants of AUUUA motifs was constructed, and 293T cells were transfected with these plasmids. Luciferase activities normalized against Renilla luciferase activities were measured to determine the binding effect of the AUUUA motifs to RPS7 in response to RPS7 overexpression or knockdown.

**Nascent RNA capture and detection**

In brief, the indicated cells were treated with 500 µM 5-ethnyluridine (EU) (Rib Bio, C10316) for 2 h to label the synthesized RNA, and then the total amount of synthesized RNA was observed using confocal microscopy based on the specific reaction between EU and Apollo® fluorescent dye. For nascent RNA capture, the EU-labeled RNAs were biotinylated with 0.25 M biotin azide (Rib Bio, C00101) in Click-iT reaction buffer, then the nascent LOXL2 RNA was measured by qRT–PCR following pull-down of biotin-conjugated, EU-labeled RNA.

**RNA decay assay**

The indicated cells were seeded into a 12-well plate, treated with actinomycin D (5 mg/mL) for 0, 1, 2, 3, 4, 5 and 6 h, and lysed with TRIzol reagent for RNA extraction. qRT–PCR analysis was performed. GAPDH served as the endogenous control. The half-life of mRNA was calculated according to the mRNA concentration and the mRNA degradation rate.

**LOXL2 enzymatic activity detection**

The LOXL2 enzymatic activity of the cell supernatant was measured using a lysyl oxidase assay kit (AAT Bioquest, 15255). Supernatant samples were prepared in buffer in a 96-well plate and incubated with the reaction mixture for 30 min according to the manufacturer’s instructions. The OD value was measured at 576 nm.

**Immunofluorescent staining**

Indicated HCC cells were planted in the cell climbing slices in a 12-well plate, fixed with paraformaldehyde, permeabilized with 0.1% TritonX-100 in PBS, and blocked with 5% BSA. Then the cell climbing slices were incubated with primary antibody (Paxillin, 1:100 ) (Abcam, ab32084) in 4℃ overnight. The following day, cell climbing slices were incubated with immunofluorescent antibodies for 1 hours at room temperature. The cells were viewed and photographed under a fluorescence microscope.

**Co-immunoprecipitation**

Briefly, indicated cells were harvested and lysed with Nonidet P-40 (NP-40) lysis buffer (20 mM Tris-HCl [pH 7.5], 150 mM NaCl, 1% NP-40). Subsequently, the scraped cell suspension was centrifuged at 13,000 rpm/min for 20 min at 4℃. Before immunoprecipitation, 2% of the extract volume was removed and served as an input. Then the supernatants were incubated with IP antibodies (LOXL2, Santa Cruz, sc-293427), incubated overnight at 4°C on a rotating wheel. Next day, after washing protein G magnetic beads (Millipore) twice with NP-40 lysis buffer, the supernatants were added and incubated with rotation for 6 h at 4°C. The bound proteins were eluted with sample buffer for SDS-PAGE followed by western blot analysis.

**Protein decay assay**

The indicated cells were seeded into a 12-well plate, treated with CHX (5 mg/mL) for 0, 8, 16, 24, 32 and 40 h, and lysed with RIPA reagent for protein extraction. Western blot analysis was performed. GAPDH served as the endogenous control. The half-life of protein was calculated according to the protein degradation rate.

**Membrane and cytosolic fractionations**

Membrane and cytosol protein were extracted from HCC cells using the Membrane and Cytosol Protein Extraction Kit (Beyotime, P0033) according to the manufacturer's instructions.

**In vivo metastasis assays**

To broadly evaluate the antimetastatic effect of RPS7 in vivo, two kinds of mouse models, an orthotopic mouse model and a lung metastasis mouse model, were constructed using RPS7-knockout MHCC97H cells. Briefly, approximately 1 × 10^6^ of the indicated cells were suspended in 20 μL serum-free DMEM/Matrigel (1:1) for each nude mouse. Through a 1 cm transverse incision in the upper abdomen under anesthesia, each nude mouse (8 in each group, 6-8 weeks old male BALB/c-nu/nu) was orthotopically inoculated in the left hepatic lobe using a microsyringe. Then, another 1 × 10^6^ cells were injected via the tail vein into nude mice (8 in each group, 6-8 weeks old male BALB/c-nu/nu). After 10 weeks, the mice were sacrificed, and their livers and lungs were dissected, fixed with 4% paraformaldehyde and prepared for HE staining. On the other hand, 2 × 10^6^ Huh7 cells stably overexpressing RPS7 were also used to construct these two mouse models, aiming to observe the influence of RPS7 overexpression on HCC cell metastasis in vivo.

In the drug intervention experiment, 2 × 10^6^ Huh7 cells stably overexpressing RPS7 were used to construct an orthotopic mouse model. Four weeks after orthotopic inoculation, intervention with 15 mg/kg CMMH (a selective inhibitor of LOXL2) by tail vein injection was implemented twice a week until the mice were sacrificed.

To furtherly investigate the effect of LOXL2 on RPS7-induced HCC metastasis, 1 × 10^6^ RPS7-knockout MHCC97H cells were used to establish orthotopic HCC models as previously described. At 2 weeks after implantation, mice were injected with AAV8-LOXL2 (1 × 10^11^ viral genomes in 100 μL saline) or AAV8-Ctrl (1 × 10^11^ viral genomes in 100 μL saline) via tail vein. Eight weeks post injection, mice were euthanized.

All mice were bred and kept under independent ventilation cage conditions. Animal studies are described according to the ARRIVE guidelines (www.nc3rs.org.uk/arrive-guidelines) and were approved by the Laboratory Animal Center of Chongqing Medical University.

**Statistical analysis**

Statistical analyses were performed with GraphPad Prism software (version 8.0). All quantitative data are expressed as the means ± standard deviation. The Mann–Whitney U test was used to analyze the data between two groups. One-way ANOVA was applied to compare data from more than two groups. The survival curves were assessed by Kaplan–Meier curves, and significant differences were evaluated by the log-rank test. The correlation of RPS7 expression with clinicopathologic parameters in HCC patients was evaluated by the chi-square test and Fisher’s exact probability method. Univariate and multivariate Cox regression analyses were performed to identify the factors that significantly affected HCC survival. P < 0.05 was considered statistically significant.

**Supplementary Table 1 Primers of different LOXL2 related fragments**

| Primer names | Sequences (5’-3’) |
| --- | --- |
| LOXL2-WT F | aaggatgacgatgacaagcttATGGAGAGGCCTCTGTGCTCCCAC |
| LOXL2-WT R | tcacagggatgccacccgggatccTTACTGCGGGGACAGCTGGTTGTTTAAGAG |
| LOXL2-Δ F | aaggatgacgatgacaagcttATGGAGAGGCCTCTGTGCTCCCAC |
| LOXL2-Δ R | tcacagggatgccacccgggatccTTAAGGGGCGGTTTCTGAGCAGGCAAC |
| LOXL2-Y689F F | ccatgggctgctgggacatgttccgccatgacatcgactgcca |
| LOXL2-Y689F R | tggcagtcgatgtcatggcggaacatgtcccagcagcccatgg |

**Supplementary Table 2 Primers of qRT-PCR in this study**

| Primer names | Sequences (5’-3’) |
| --- | --- |
| RPS7 F | TCTTTGTTCCCGTTCCTCAACT |
| RPS7 R | AGCTGTCAGAGTACGGCTCCT |
| LOXL2 F | CTCCACTGTACTGGCAACGA |
| LOXL2 R | GCGGTAGGTTGAGAGGATGG |
| ITGB1 F | AATGTAACCAACCGTAGCA |
| ITGB1 R | CTGAAGTCCGAAGTAATCCT |
| β-actin F | CTCTTCCAGCCTTCCTTCCT |
| β-actin R | AGCACTGTGTTGGCGTACAG |
| GAPDH F | TATGACAACAGCCTCAAGAT |
| GAPDH R | AGTCCTTCCACGATACCA |

**Supplementary Table 3 Primers of RNA pull-down in this study**

| Primer names | | Sequences (5’-3’) |  |
| --- | --- | --- | --- |
| LOXL2 5' UTR | F | TAATACGACTCACTATAGGGTACCTACGCTTGGTGCTT | |
|  | R | TGTCTTCGGGCTGATGAT | |
| LOXL2 CDS | F | TAATACGACTCACTATAGGGCAGAAGAGGAAGCACAG | |
|  | R | AAGTTGGCACACTCGTAAT | |
| LOXL2 3' UTR | F | TAATACGACTCACTATAGGGAGAAGCCTGCGTGGTCAACT | |
|  | R | GTTTCAGTAAAAACCACAGG | |
| LOXL2  (2576-2975) | F | TAATACGACTCACTATAGGGagaagcctgcgtggtcaact | |
|  | R | ctgatgagcccgcatttgtc | |
| LOXL2  (2976-3375) | F | TAATACGACTCACTATAGGGcccatttctcctcctcttag | |
|  | R | aggaccctggttatagcacc | |
| LOXL2  (3376-3721) | F | TAATACGACTCACTATAGGGccatgtgtcatcacagacac | |
|  | R | ctccttagattgcttctccc | |
| LOXL2  (2976-3175) | F | TAATACGACTCACTATAGGGtcaggccgaaccccatttct | |
|  | R | gaaaaacaaggggtgg | |
| LOXL2  (3155-3375) | F | TAATACGACTCACTATAGGGgaaaaacaaggggtgg | |
|  | R | acccaggaccctggttatag | |
| LOXL2  (3190-3259)-WT |  | TAATACGACTCACTATAGGGcacagacttttgaagcacaaatttattggcatttaatattggacatctgg | |
| LOXL2  (3190-3259)-Mut1 |  | TAATACGACTCACTATAGGGcacagacttttgaagcacaatatatttggcatttaatattggacatctgg | |
| LOXL2  (3190-3259)-Mut2 |  | TAATACGACTCACTATAGGGcacagacttttgaagcacaaatttattggctatatatattggacatctgg | |
| LOXL2  (3190-3259)-Mut3 |  | TAATACGACTCACTATAGGGcacagacttttgaagcacaatatatttggctatatatattggacatctgg | |

**Supplementary Table 4 Primers of dual luciferase reporter assay in this study**

| Primer names |  | Sequences (5’-3’) |
| --- | --- | --- |
| LOXL2-promoter  （-1651/+122） | F | CTAGCTAGCGTGAGATGTGGTTGGCAGTT |
|  | R | CCCAAGCTTCACCAAGCGTAGGTAGCC |

**
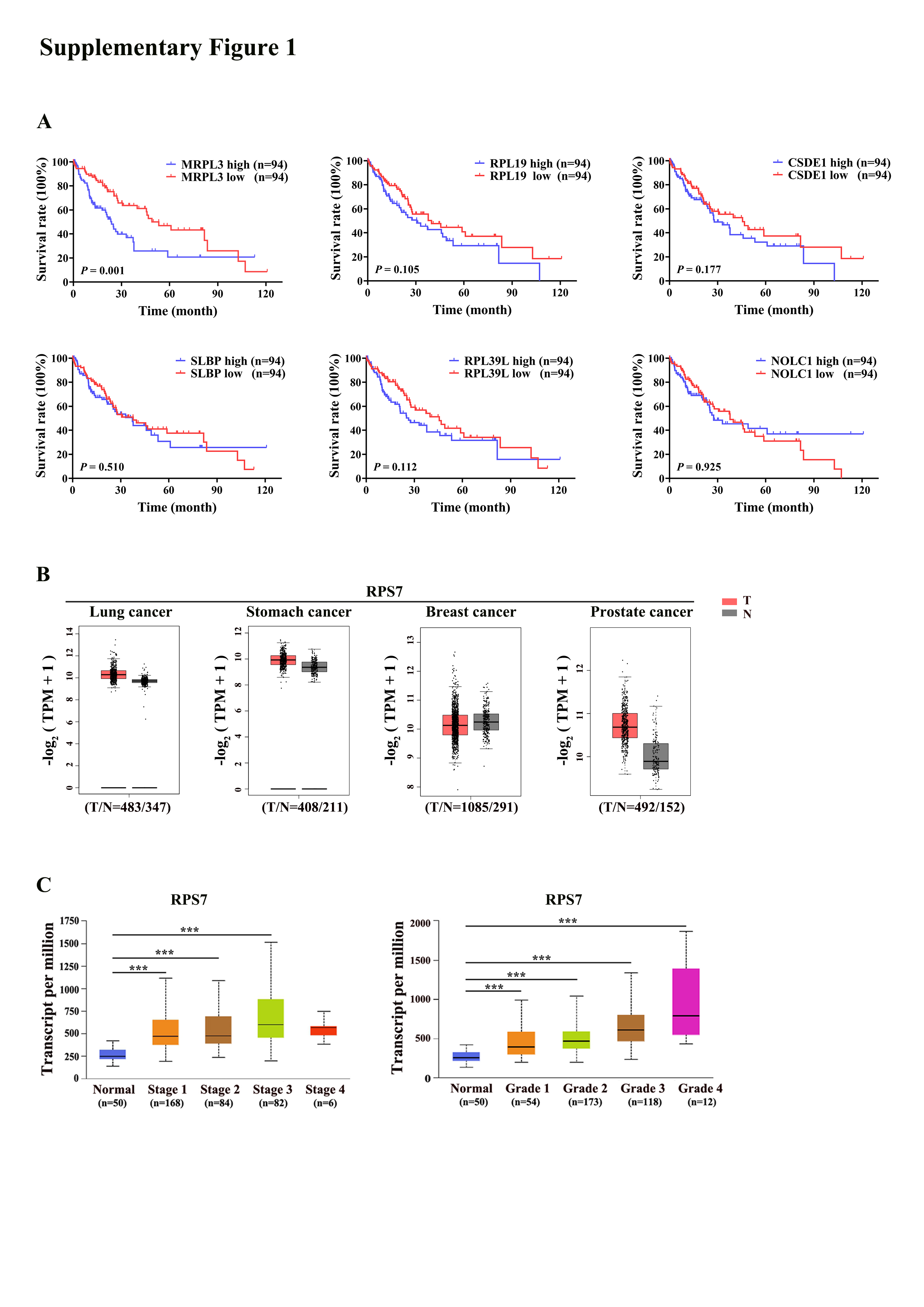
**

**Supplementary Fig.1 Identification of RPS7 as an important gene closely associated with HCC progression.** A. The correlation between expression levels of each gene and overall survival rate in HCC with metastases. B. The differential expression of RPS7 in other frequent cancers except HCC. C. The correlation between RPS7 expression and HCC histological grades and clinical stages, respectively. ***, *P* < 0.001.


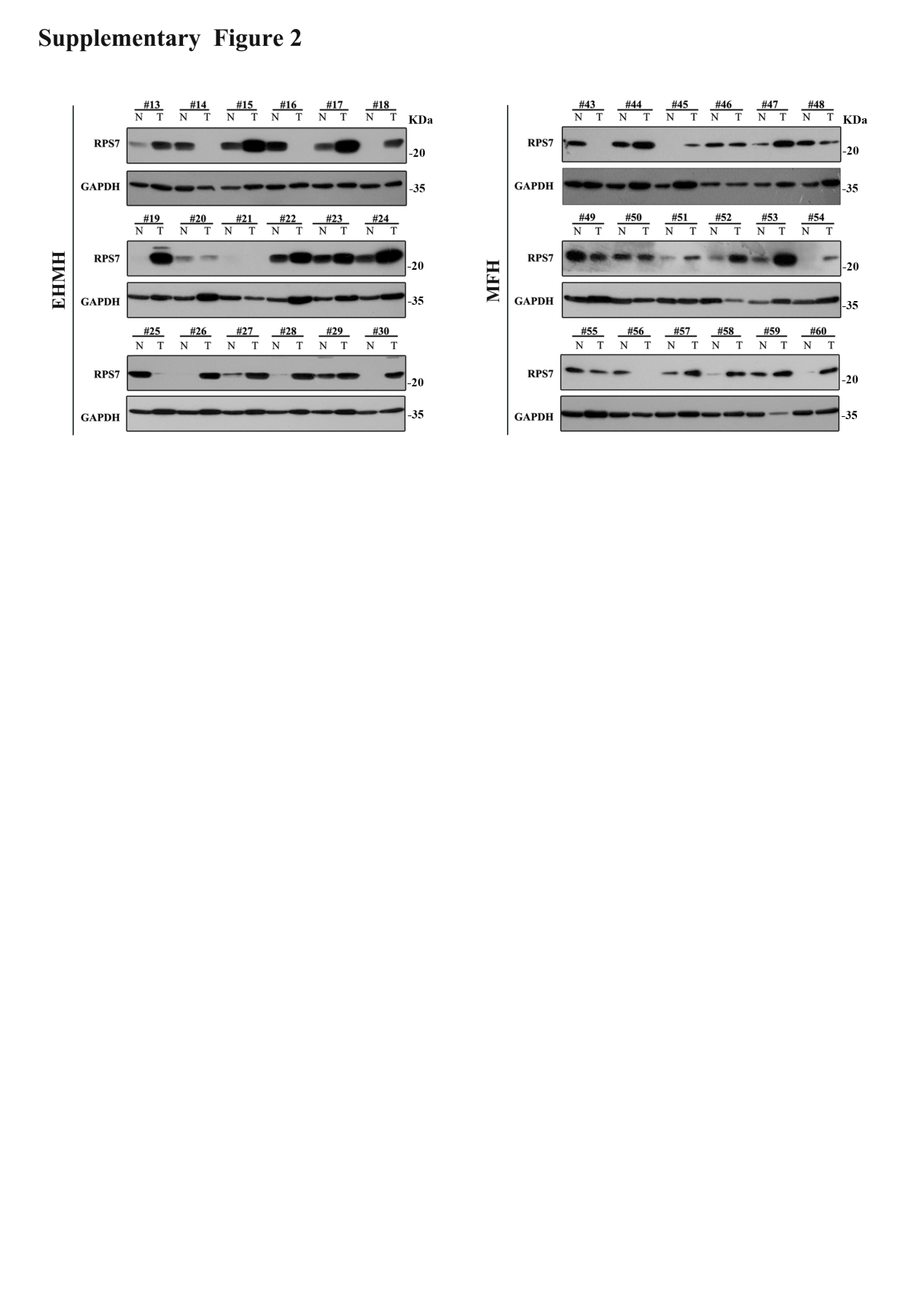


**Supplementary Fig.2 The expression of RPS7 in HCC tissues and matched normal liver tissues.** Western blot was performed to detect RPS7 protein levels in EHMH group and MFH group, respectively. GAPDH was used as loading control. EHMH, HCC tissues with extrahepatic metastasis; MFH, metastasis-free HCC tissues.

**
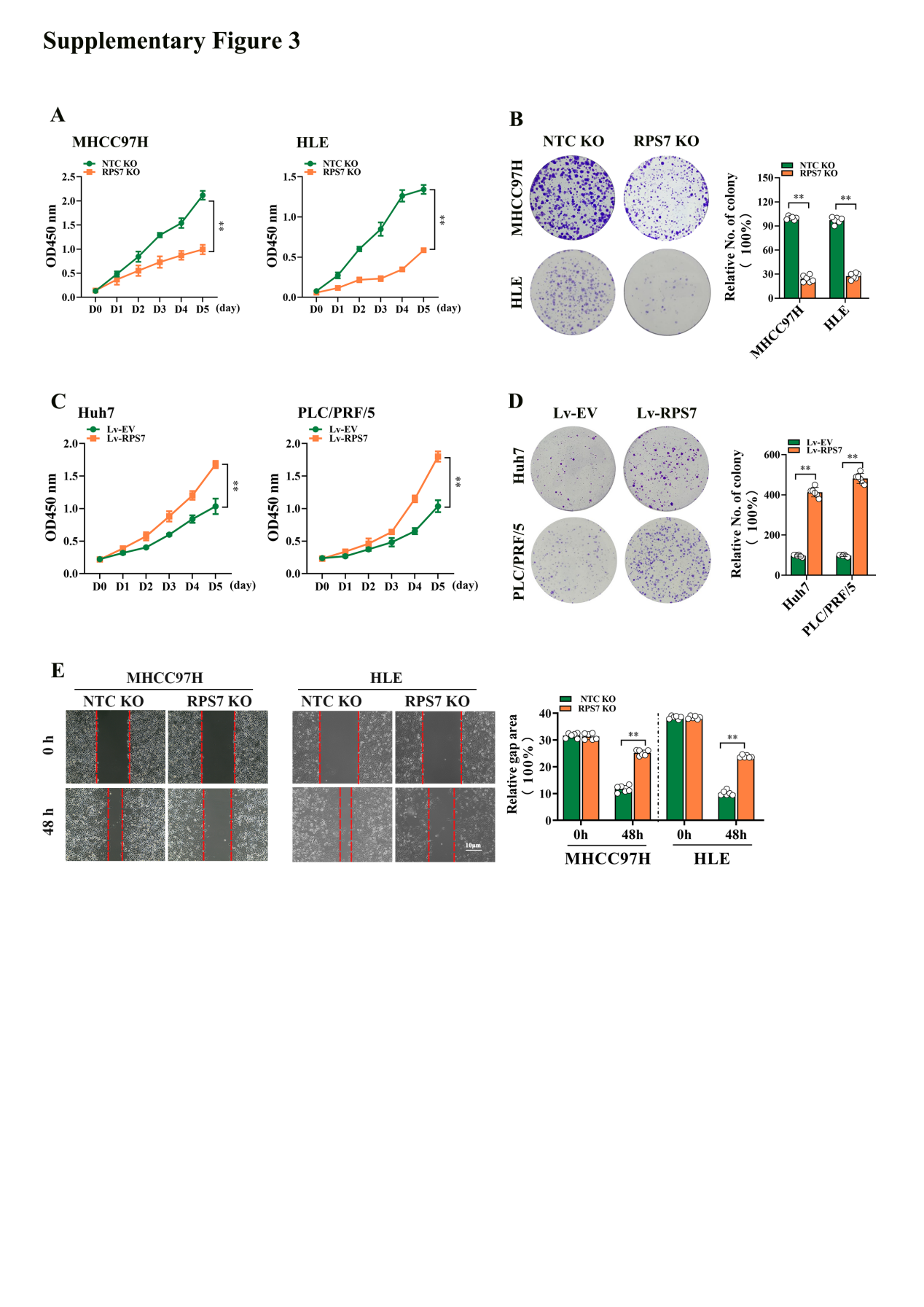
**

**Supplementary Fig.3 Effect of RPS7 on HCC cell phenotypes in vitro.** A and B. Effect of PRS7 knockout on MHCC97H and HLE proliferation was determined by CCK-8 assay (A) and colony formation assays (B). C and D. Effect of RPS7 overexpression on Huh7 and PLC/PRF/5 cells proliferation was determined by CCK-8 assay (C) and colony formation assays (D). E. The wound closure abilities of RPS7-knockout cells were determined by wound healing assay. Representative data are from at least 3 independent experiments. Data are shown as mean ± SD. **, *P* < 0.01.

**
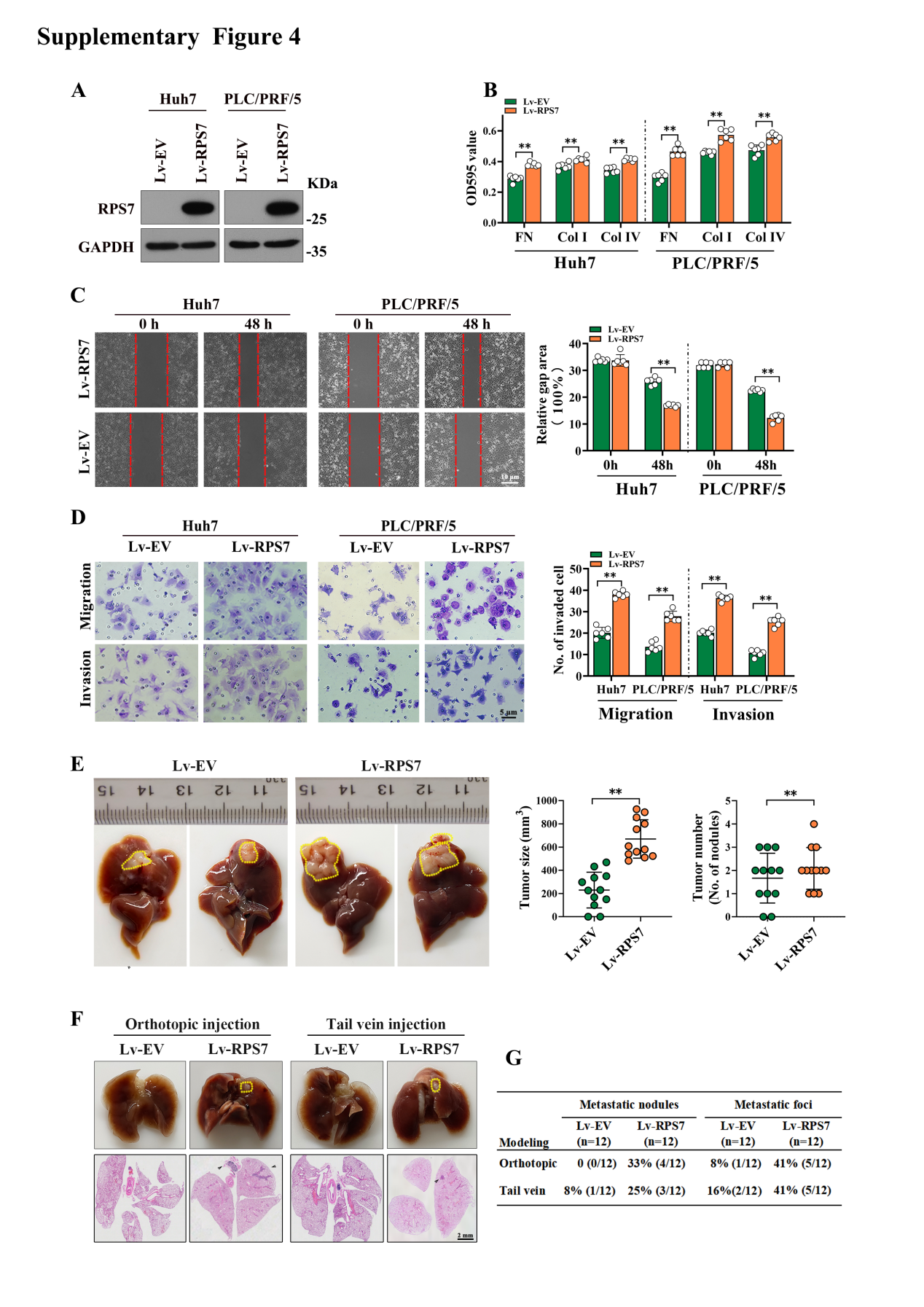
**

**Supplementary Fig.4 Overexpression of RPS7 promotes HCC cell adhesion, migration and invasion in vitro and metastasis in vivo.** Huh7 and PLC/PRF/5 cells, two poorly aggressive HCC cell lines, were used to establish stable RPS7 overexpression cells via lentivirus carrying RPS7 (Lv-RPS7). Cells infected with lentivirus carrying empty vector were correspondingly used as controls (Lv-EV). The cell-matrix adhesion capacity, migration and invasion ability of cells in vitro as well as metastasis in vivo were observed under RPS7 overexpression. A. Overexpression efficiency was determined by western blot assay. B. RPS7-overexpressed HCC cells adhesion to fibronectin, collagen I and collagen IV were detected using cell-matrix adhesion assay. C. The wound closure abilities of RPS7-overexpressed cells were determined by wound healing assay. D. The effect of RPS7-overexpressed on cell migration and invasion were determined by Transwell assay. E. A orthotopic mouse model was constructed using RPS7-overexpressed Huh7 cells and control cells (each group, n=12). The effect of RPS7-overexpressed on tumor size and numbers were evaluated. F and G. The effects of RPS7-overexpressed on lung metastasis were evaluated by orthotopic mouse models and tail vein lung metastasis mouse models, respectively. Representative data are from at least 3 independent experiments. Data are shown as mean ± SD. *, *P* < 0.05, **, *P* < 0.01.

**
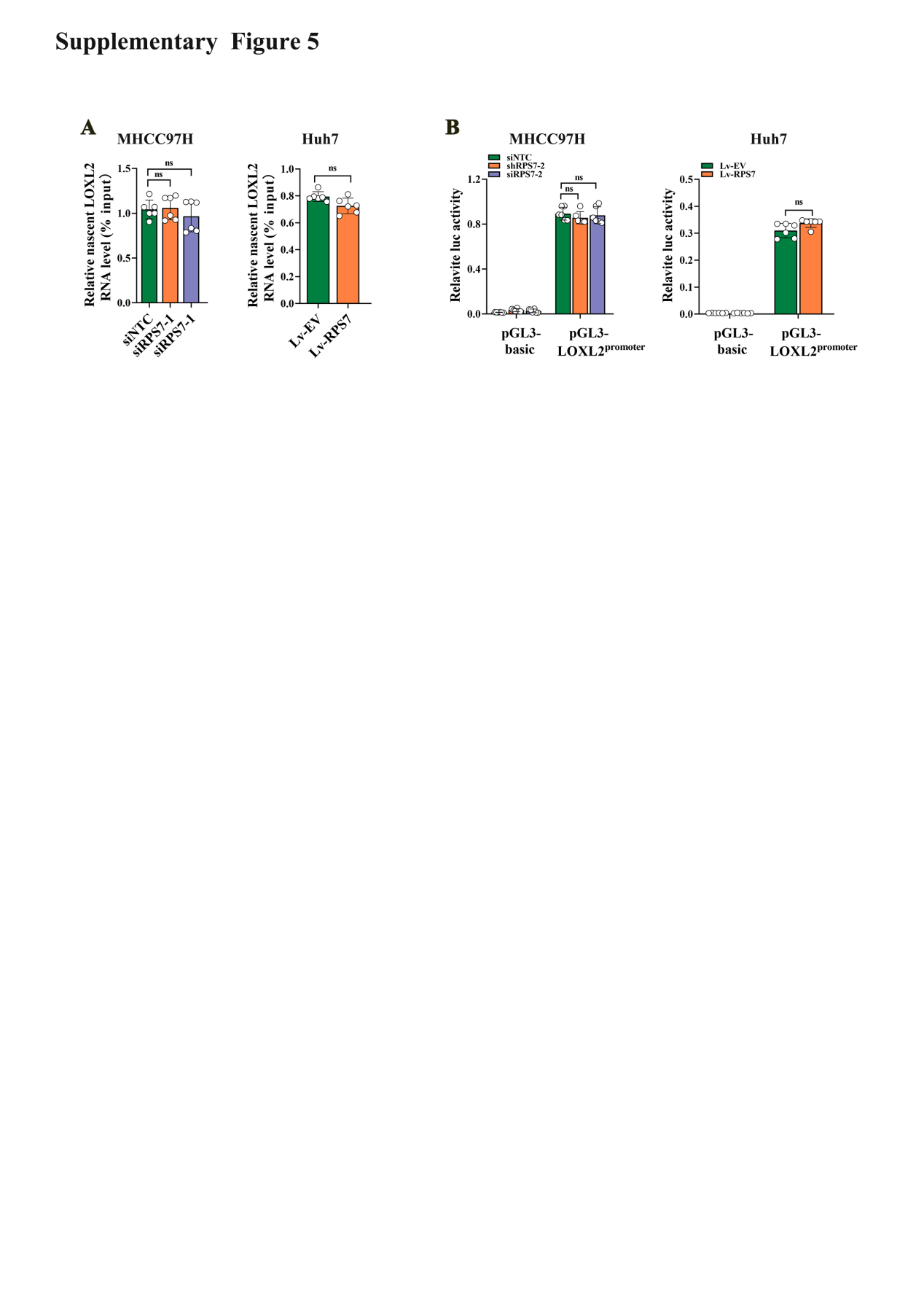
**

**Supplementary Fig.5 Effect of RPS7 on LOXL2 expression at transcriptional level**. A. Effect of RPS7 overexpressing/knockdown on LOXL2 promoter activity was detected using dual luciferase reporter assay, respectively. B. Nascent LOXL2 RNA was measured by qRT-PCR following pull-down of biotin-conjugated, EU-labeled RNA in RPS7 knockdown or overexpressed HCC cells. Representative data are from at least 3 independent experiments. Data are shown as mean ± SD. ns, no significant.

**
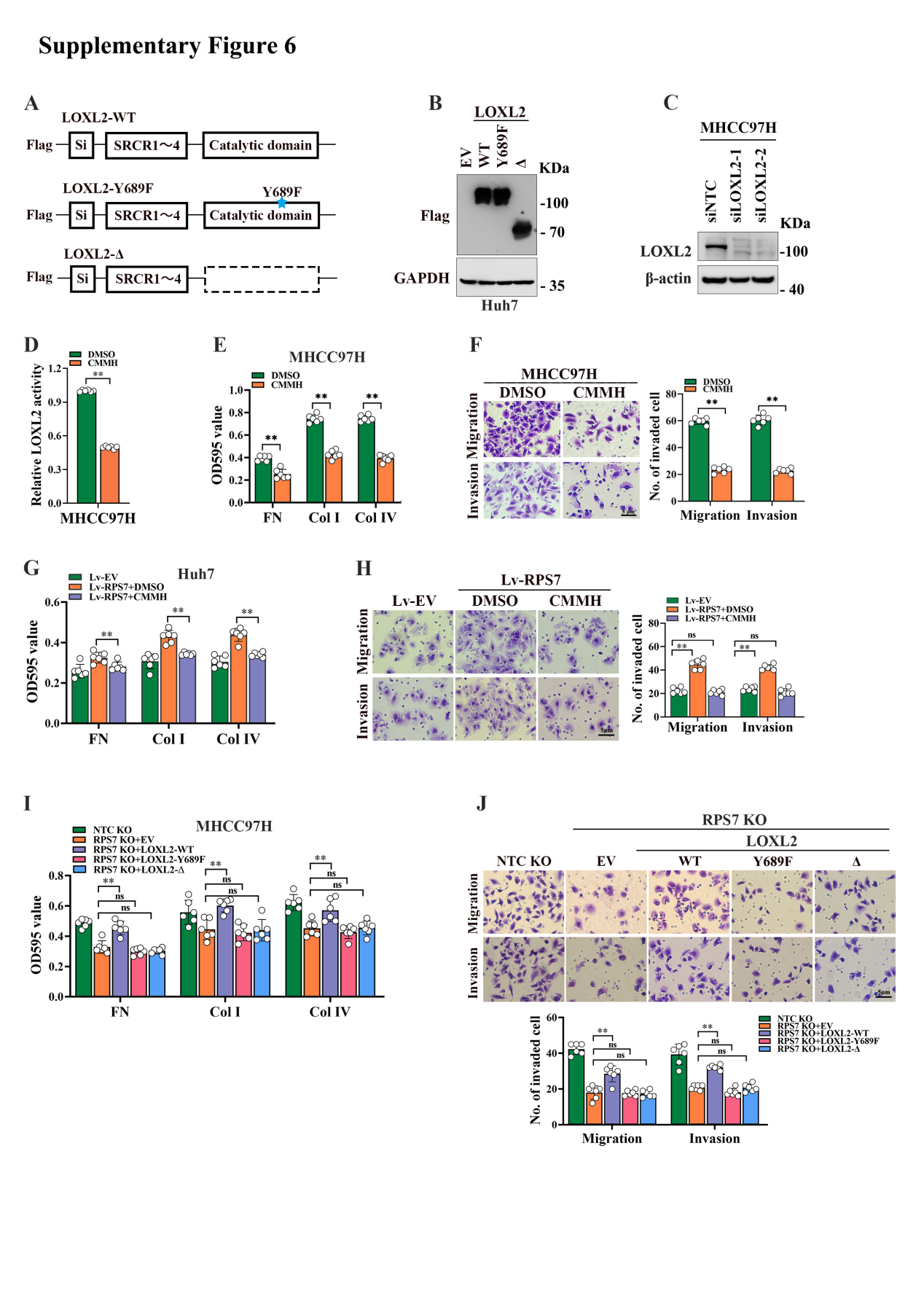
**

**Supplementary Fig.6 LOXL2 mediates RPS7-induced cell adhesion, migration and invasion.** A. Catalytic activity deletion mutant of LOXL2 (LOXL2-Δ) and catalytically inactive point mutant of LOXL2 (LOXL2-Y689F), as well as wild type LOXL2 (LOXL2-WT) were respectively constructed. B. Such plasmids were transfected into Huh7 cells. Effect of LOXL2 overexpression was determined by western blotting. C. Effect of LOXL2 silencing was determined by western blotting. D. The impact of 20 μM CMMH on LOXL2 enzyme activity was evaluated using a lysyl oxidase assay kit. MHCC97H cells were treated with 20 μM CMMH, a highly selective LOXL2 enzyme inhibitor, the effect of CMMH on cell-matrix adhesion ability (E) and migration and invasion (F) were analyzed. G. Cell-matrix adhesion assay was performed to evaluate the effect of 20 μM CMMH on the adhesion ability of RPS7-overexpressed Huh7 cells. H. Transwell assay was performed to assess the effect of 20 μM CMMH on the migration and invasion abilities of RPS7-overexpressed Huh7 cells. I. Cell-matrix adhesion assay was performed to evaluate the effect of LOXL2 overexpressing on the adhesion ability of RPS7-knockout MHCC97H cells. J. Transwell assay was performed to assess the effect of LOXL2 overexpressing on the migration and invasion abilities of RPS7-knockout MHCC97H cells. Data are shown as mean ± SD. **, *P* < 0.01. ns, no significant.

**
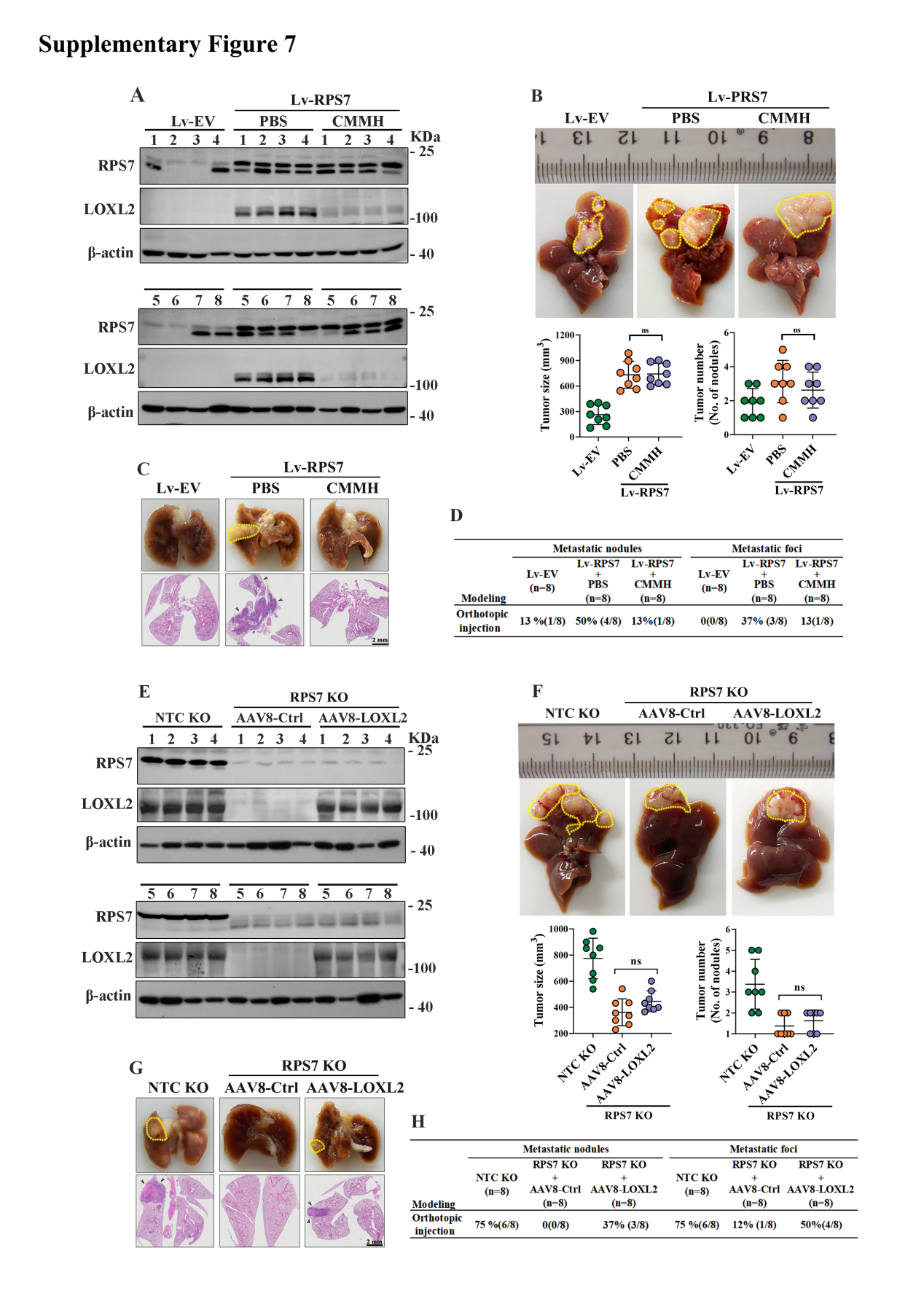
**

**Supplementary Fig.7 LOXL2 is involved in RPS7-mediated HCC metastasis in vivo.** A-D. RPS7-overexpressed Huh7 cells were orthotopically injected into the left lobe of orthotopic liver of the nude mice. Four weeks after inoculation, CMMH (15 mg/kg) was administered twice a week for six weeks through tail vein injection. Expression of LOXL2 and RPS7 in tumor tissues were detected by western blot (A). Tumor size and numbers were evaluated in CMMH-treated mice compared to control group (B). Lung metastasis were evaluated according to detect metastatic nodules and foci (C and D). E-H. Orthotopic HCC models were established by using RPS7-deletion MHCC97H cells. At 2 weeks after implantation, mice were injected with AAV8-LOXL2 (1 × 10^11^ viral genomes in 100 μL saline) or AAV8-Ctrl (1 × 10^11^ viral genomes in 100 μL saline) via tail vein. Expression of LOXL2 and RPS7 in tumor tissues were detected by western blot (E). Tumor size and numbers were evaluated between each group (F). Lung metastasis were evaluated according to detect metastatic nodules and foci (G and H). Representative data are from at least 3 independent experiments. Data are shown as mean ± SD. **, *P* < 0.01. ns, no significant.

**
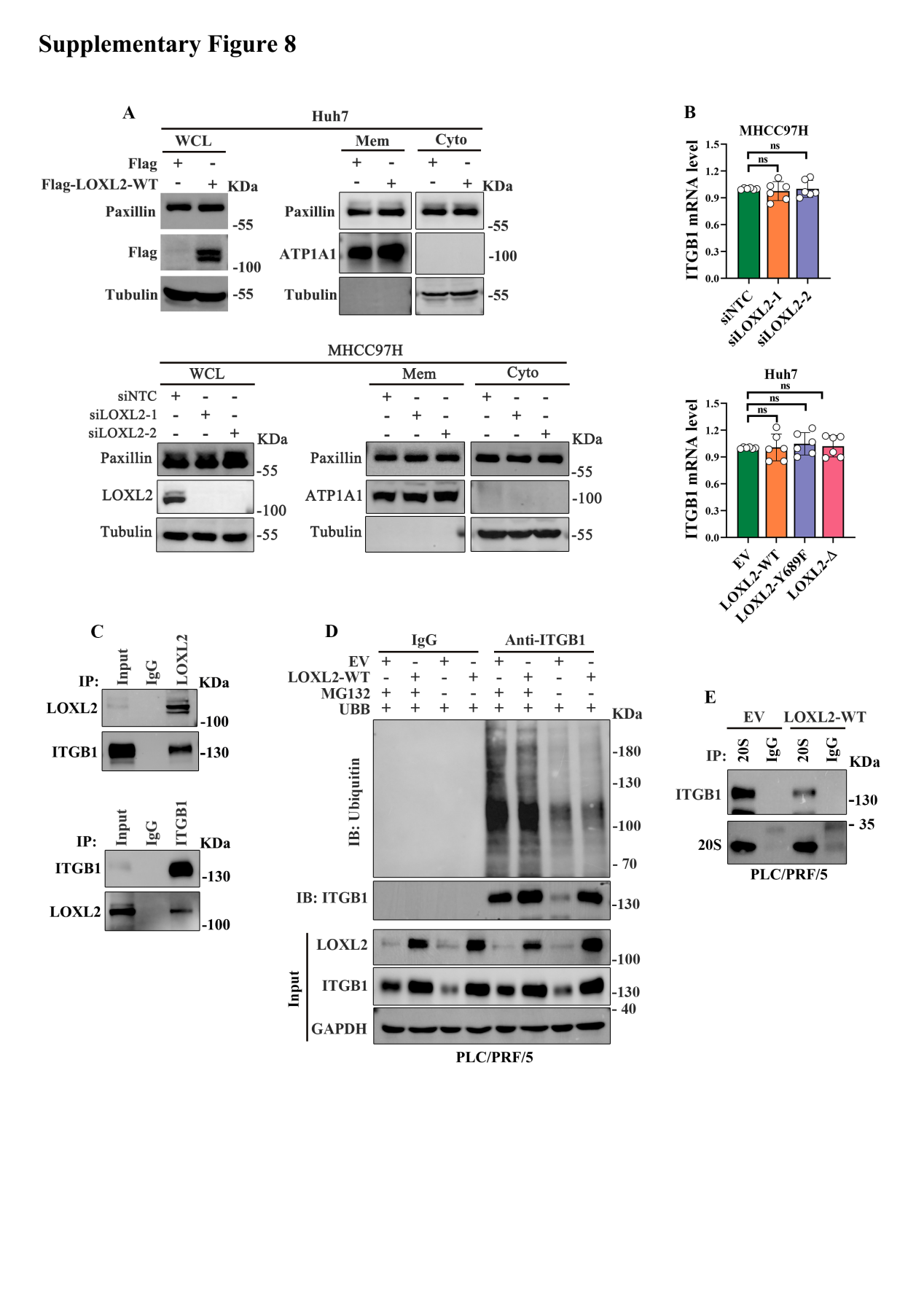
**

**Supplementary Fig.8 LOXL2 regulates ITGB1 expression at protein level.** A. Membrane and cytosolic fractionations was performed to explore whether LOXL2 alterations affects the levels of membrane-associated Paxillin. B. qRT-PCR was performed to detect the influence of LOXL2 on ITGB1 mRNA level. C. Co-immunoprecipitation (co-IP) assay was performed to in HLE cells to evaluate the interact between LOXL2 and ITGB1. D. Whole-cell extracts of LOXL2 overexpressed PLC/PRF/5 cells were immunoprecipitated with anti-ITGB1 antibody, and ubiquitinated ITGB1 was detected with anti-ubiquitin antibody. E. The immunoprecipitation of 20S and ITGB1 was detected in PLC/PRF/5 cells transfected with empty vector or LOXL2-WT plasmids. Data are shown as mean ± SD. ns, no significant.


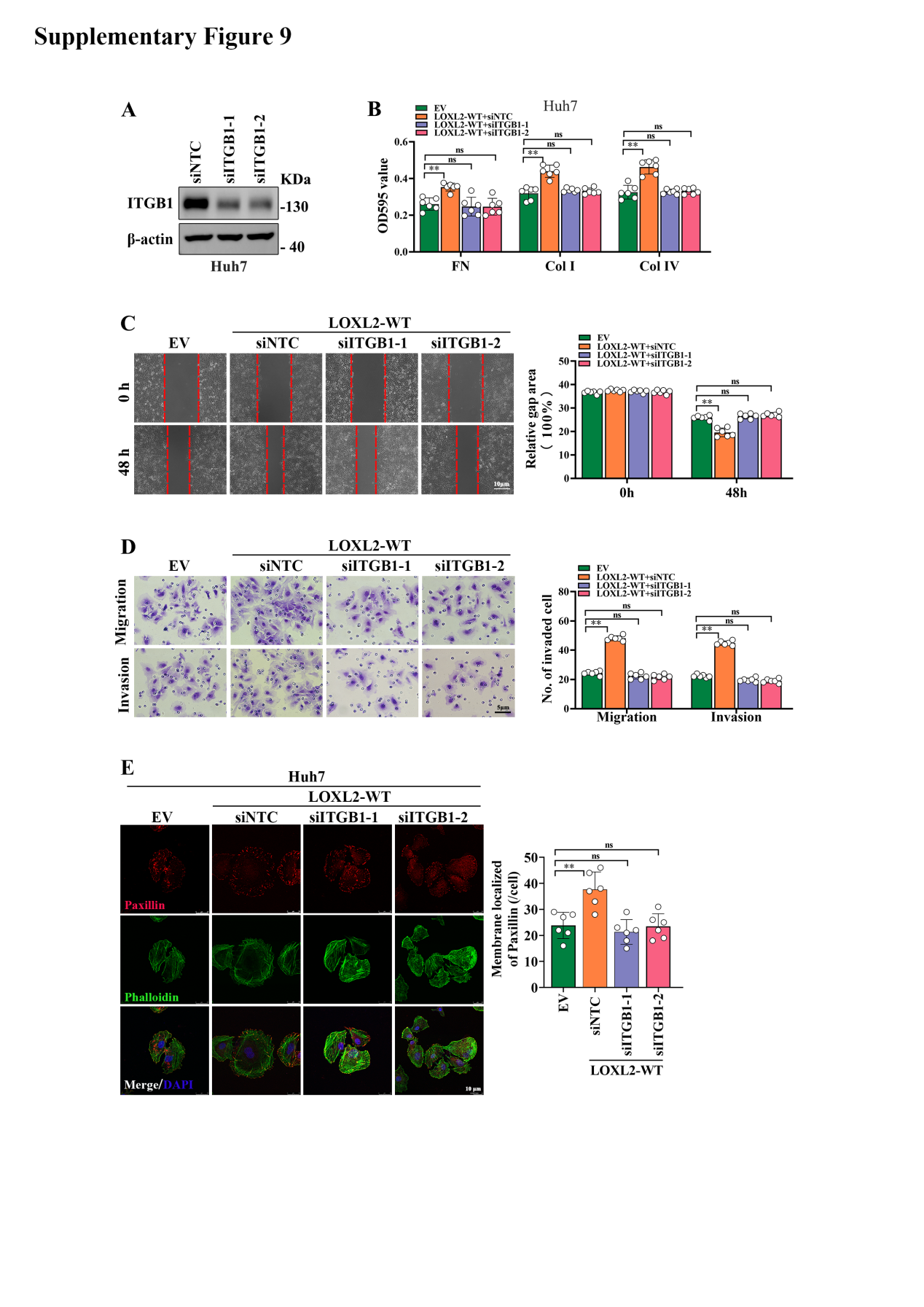


**Supplementary Fig.9 ITGB1 is involved in LOXL2-mediated malignant phenotypes of HCC cells**. A. The effect of ITGB1 silencing in Huh7 cells was determined by western blotting. B-E. Functional cell experiments were performed to evaluate the effect of ITGB1 on LOXL2-overexpressed Huh7 cells adhesion capacity (B), wound closure ability (C), migration and invasion abilities (D) and focal adhesion formation (E). Representative data are from at least 3 independent experiments. Data are shown as mean ± SD. **, *P* < 0.01. ns, no significant.


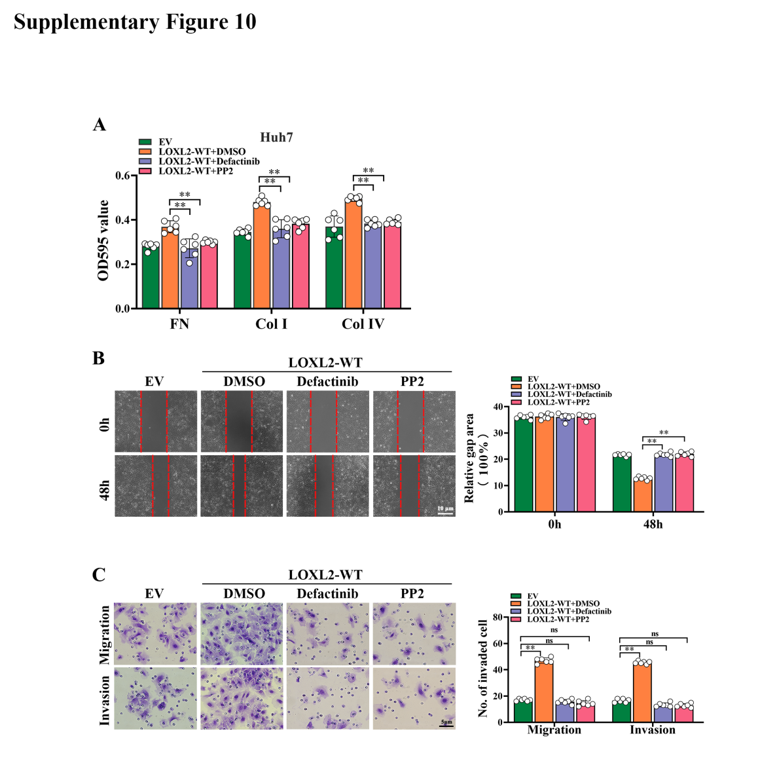


**Supplementary Fig.10 FAK/SRC signaling is involved in LOXL2-mediated invasive phenotype of HCC cells.** LOXL2-overexpressed Huh7 cells were treated with Defactinib, a FAK inhibitor and PP2, a SRC inhibitor, for 48h, respectively. Functional cell experiments were performed to evaluate the effect of such inhibitors on cells adhesion capacity (A), wound closure ability (B), migration and invasion abilities (C). Representative data are from at least 3 independent experiments. Data are shown as mean ± SD. **, *P* < 0.01. ns, no significant.


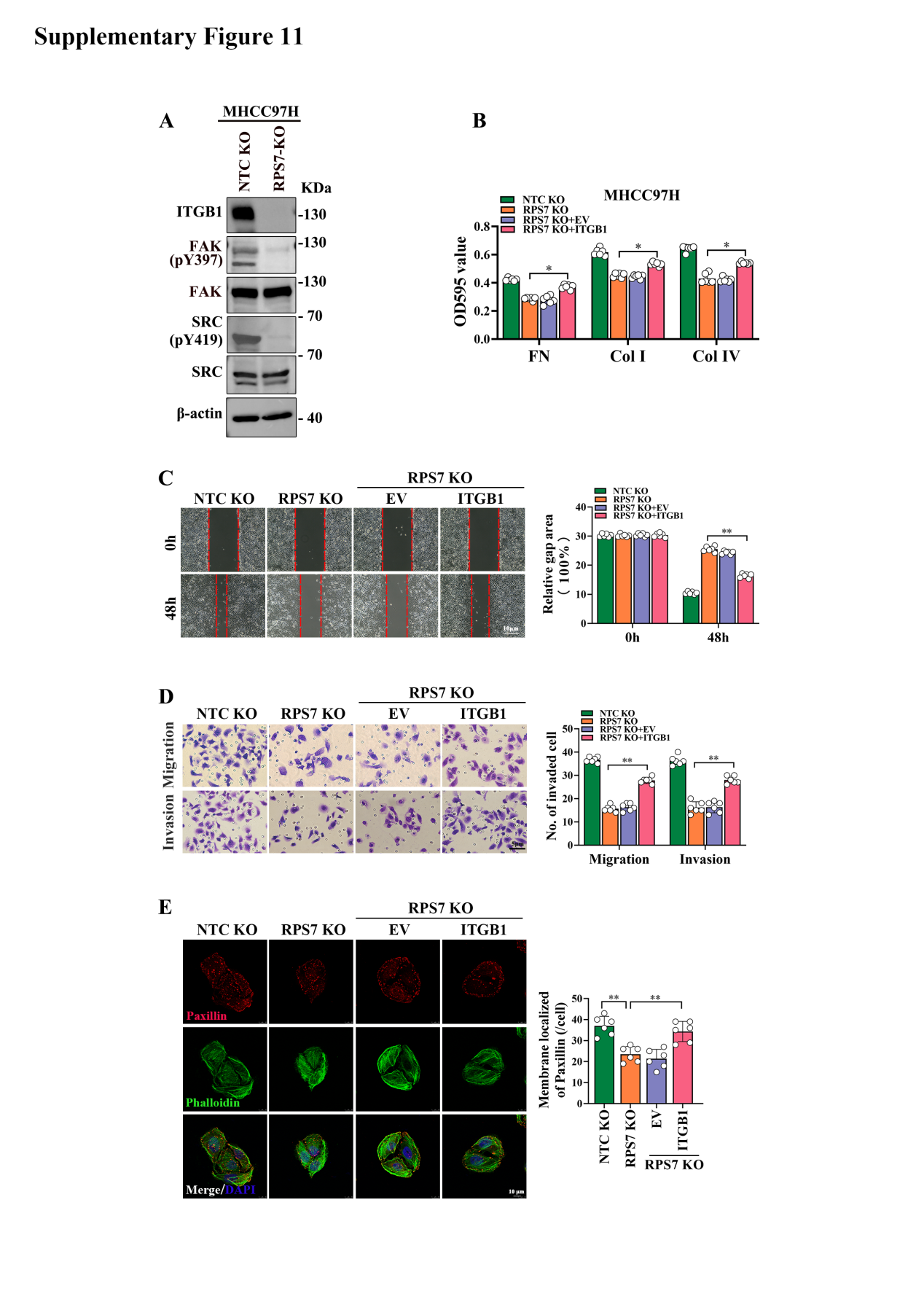


**Supplementary Fig.11 ITGB1 is involved in RPS7-mediated invasive phenotype of HCC cells.** A. The effect of RPS7 knockout on signal activity of ITGB1/FAK/SRC pathway was determined by western blotting. B-E. Functional cell experiments were performed to evaluate the effect of ITGB1 on RPS7-knockout MHCC97H cells adhesion capacity (B), wound closure ability (C), migration and invasion abilities (D) and focal adhesion formation (E). Representative data are from at least 3 independent experiments. Data are shown as mean ± SD. *, *P* < 0.05, **, *P* < 0.01.
